# A Stretchable and Self-Healing Hybrid Nano-Generator for Human Motion Monitoring

**DOI:** 10.3390/nano12010104

**Published:** 2021-12-29

**Authors:** Yongsheng Zhu, Fengxin Sun, Changjun Jia, Tianming Zhao, Yupeng Mao

**Affiliations:** 1 Physical Education Department, Northeastern University, Shenyang 110819, China; 2001276@stu.neu.edu.cn (Y.Z.); 2171435@stu.neu.edu.cn (F.S.); 2071367@stu.neu.edu.cn (C.J.); 2 College of Sciences, Northeastern University, Shenyang 110819, China

**Keywords:** self-powered, sports monitoring, hydrogel, hybrid nano-generator

## Abstract

Transparent stretchable wearable hybrid nano-generators present great opportunities in motion sensing, motion monitoring, and human-computer interaction. Herein, we report a piezoelectric-triboelectric sport sensor (PTSS) which is composed of TENG, PENG, and a flexible transparent stretchable self-healing hydrogel electrode. The piezoelectric effect and the triboelectric effect are coupled by a contact separation mode. According to this effect, the PTSS shows a wide monitoring range. It can be used to monitor human multi-dimensional motions such as bend, twist, and rotate motions, including the screw pull motion of table tennis and the 301C skill of diving. In addition, the flexible transparent stretchable self-healing hydrogel is used as the electrode, which can meet most of the motion and sensing requirements and presents the characteristics of high flexibility, high transparency, high stretchability, and self-healing behavior. The whole sensing system can transmit signals through Bluetooth devices. The flexible, transparent, and stretchable wearable hybrid nanogenerator can be used as a wearable motion monitoring sensor, which provides a new strategy for the sports field, motion monitoring, and human-computer interaction.

## 1. Introduction

The Chinese diving team won gold (silver) medals in the men’s and women’s ten-meter platform events in the Tokyo Olympic Games in 2020. This excellent performance is related to “Rip entry” technology. Athletes’ entry into the water is a fluid-solid coupling problem [1,2,3,4]. This technology helps athletes to reduce the impact force when their hands enter into the water in a square shape. On the contrary, when athletes’ hands enter the water in a wedge shape, the water shows the characteristic of escaping in the direction of lower pressure. The larger the wedge angle, the higher the wave height of the free liquid surface and the greater the splash [5,6]. Therefore, athletes must change their hands into a square shape immediately before entering the water [7]. In other words, athletes need to complete this technology according to the direction of rotation, the folding speed of the palms, and the orientation of the palms. “Rip entry” motion is not a simple process of moving the palms inward. In the process of training, this technology should be scientifically monitored by coaches and athletes, in order to learn high-quality movements. At present, some researchers have been studying “Rip entry” technology in sports biomechanics theory [8,9,10]. However, the monitoring of this technology is still an isolated field. First of all, the palms and finger joints are small joints, and a single high-speed camera cannot capture the motion at all [11,12,13]. In addition, athletes’ palms need to remain in a square shape before entering the water. Recent studies show that most wearable sensors can only bend in the longitudinal axis [14,15]. It is necessary to improve the flexibility and stretchability of these sensors for twist motions. Meanwhile, capacitor/resistance sensors present a risk of electricity leaking when the sensors are in the water [16,17,18,19,20,21]. Therefore, a soft, flexible, and self-powered sensor needs to be developed which can be used to monitor “Rip entry” technology. Moreover, this would also be practical for the scientific training of diving events and would provide some new ideas for other sports technology monitoring such as backhand twisting and pulling action in table tennis, shot put, and so on.

In recent years, more and more attention has been paid to health and sports. With the development of IOTs and big data, a large number of flexible intelligent motion monitoring devices have been integrated. Wang’s group created TENG (triboelectric nano-generator) [22,23,24] and PENG (piezoelectric nano-generator) [25,26]. These devices possess excellent working performance, a simple structure, long-life advantages, and are low cost [27,28,29,30,31,32,33,34,35]. Some researchers have coupled TENG and PENG together [36,37,38,39,40,41,42]. This can transfer the pressure, frequency, and acceleration of irregular and low frequency body motions signal to electronic equipment to achieve wireless transmission. Meanwhile, researchers have considered how TENG and PENG work underwater and the studies have shown that they have a good design structure and function [43,44,45,46,47]. However, the devices are big for body motion monitoring and they have rigid structures.

In this study, a self-powered sensor is prepared which is coupled with PENG and TENG. PVDF is used as the sensitive layer of PENG. TENG adopts the single-electrode method, transparent PTFE is the negative electrode layer of TENG, and the skin is the positive electrode layer. The transparent PTFE and PVDF use the common electrode and the PTFE is not only the negative layer of TENG, but also the protective layer of PENG. Through this combined mode, power generation performance is improved largely. PTSSs can convert mechanical energy into electrical energy, which provides more possibilities for carbon neutralization and peak emission of carbon dioxide. It is noted that hydrogel is used as the electrode of the sensor. As a flexible electrode, the hydrogel has the following characteristics: stretchability, lightweight, small size, high transparency, good biocompatibility, simple production, and low cost. Considering the comfortability of sport monitoring and complex body motion characteristics, the sports monitoring sensor which is composed of a coupling device of TENG, PENG, and stretchable hydrogel electrodes, can be used for body sport training and health monitoring to develop more intelligent and comfortable applications.

## 2. Materials and Methods

### 2.1. Materials

Poly (vinylidene fluoride) (PVDF) powder was bought from Qinshang plastic Co., Ltd. (Suzhou, China). N, N-Dimethylformamide (DMF), deionized water, Acrylamide (AM), Lithium chloride (LiCl), N′,N′-methylene diacrylamide (MBA), Ammonium persulphate (APS), and N′,N′,N′,N′-Tetramethylethylenediamine (TMLD) were bought from Jintong letai chemical industry products Co., Ltd. (Beijing, China). Dow Corning 3140 RTV and Svlgard 184 were bought from Xinheng trading Co., Ltd. (Tianjin, China). The transmittance PTFE was bought from Taizhou huafu plastic industry Co., Ltd. (Taizhou, China). The latex was bought from Ji’nan Chuangyuan Chemical Co., Ltd. (Jinan, China).

### 2.2. Preparation

**Figure 1:**
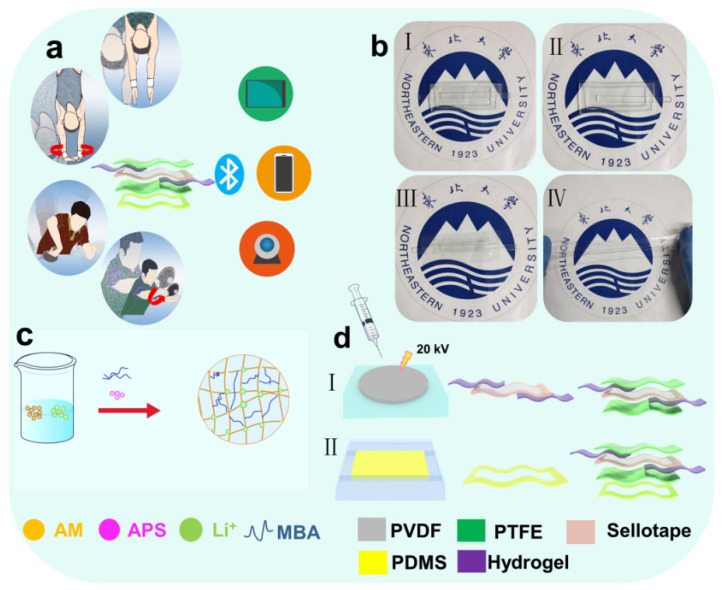
The drawing of the PTSS and its applications. (**a**) Scene of how the PTSS is used for sports. (**b**) Optical image of the PTSS (**I**), TENG (**II**), PENG (**III**), and hydrogel (**IV**). (**c**) Manufacturing step of the hydrogel. (**d**) Manufacturing step of the PVDF film and PDMS substrate and schematic diagram of the PTSS combination.

Fabrication of hydrogel: AM as monomer, MBA as cross-linking agent, APS as initiator, and TMED as catalyst. Firstly, 12 g acrylamide powder and 14 g lithium chloride particles were added in 40 mL pure water to stir using a magnetic stirrer, the magnetic stirrer was set to 600 revolutions per minute. Furthermore, the 4.23 mol/L and 8.24 mol/L of AM and LiCl mixture solution were prepared, respectively. Secondly, after stirring for 5 min, the 0.04 mol/L APS and 0.03 mol/L MBA were added to the mixture solution to be stirred together for 5 min, following this it should stand for 5 min to get the pre solution. Thirdly, three drops of TMEDA were dropped in the pre solution and stirred for 2 min to accelerate commissure for the hydrogel. Finally, the mixture solution was dropped on the culture plate to gain PAAM-LiCl hydrogel (Figure 1c).

Fabrication of the PVDF film: Firstly, the 15 wt% P(VDF-TrFE) polymer powder and 85 wt% DMF solution were stirred in a water bath at 60 °C for 4 h. For removing bubbles, semitransparent mixture solution was set in a vacuum drying oven for 30 min. Secondly, the mixed solution was dropped on the bottom silicon rotating surface of the spin coater, which was rotated at 400 rpm for the 20 s. Then, the PVDF/MBA mixture was dried at 80 °C for 15 min. After the second step was repeated three times, the mixture solution was dried at 120 °C for 12 h, and the multilayer film was prepared (Figure 1(dI)).

Fabrication of the PDMS substrate: Firstly, the PDMS solution and solidifying agent were mixed together with a 10:1 weight ratio for 3 min. Secondly, the mixture solution was stirred by an ultrasonic stirrer for 5 min. Thirdly, after being stirred for 5 min, we dropped it on the mold and we put it in the heating furnace at 80 °C for 10 min to get the PDMS substrate film. Finally, the PDMS was cut according to our needs (Figure 1(dII)).

Fabrication of latex film: The latex solution was poured into the glass mold and dried at room temperature for 24 h to get latex film with a thickness of 2 mm.

Fabrication of the whole sensor: Firstly, the PVDF edges were sealed with insulating tape to avoid short-circuiting. This is because when the hydrogel is applied on the PVDF film, the edges of the film are very thin, and if there is no anti-short circuit treatment, the hydrogel contacts with double sides and it can cause short-circuiting. Secondly, double hydrogel electrodes were covered on both sides of the PVDF and then both sides were tightly fixed by transmittance PTFE films. The PENG was packaged by Dow Corning 3140 RTV. Therefore, the hydrogel could not dehydrate in a short time, as this improves the stability and extends its service life. Thirdly, the PMDS was hollowed out at middle position to let the skin contact the PTFE. It was noted that the PDMS should be fixed to the negative dipole side of the PVDF to allow the TENG and PENG to couple together. In the basic test, the latex film was attached to the PMDS substate to replace the skin. In the practical test, the latex was removed and skin contacted the PEFE directly.

### 2.3. Characterization and Measurement

The PTSS was fixed on the stepping motor to simulate joint movement. The different amplitudes and frequencies were used to test PTSS characteristics. Sensing signals were generated by the PTSS and collected by oscilloscopes (sto1102c, Shenzhen, China). The stress-strain test was carried out by an optical microscope (Scientific compass Co., Ltd., Cmt6103 manufacturer MTS Mets industrial system, Shenyang, China).

## 3. Results

Diving belongs to the category of difficult and beautiful events The motions of diving are fast and subtle; these motions cannot be captured and analyzed by sports monitoring equipment easily. However, the PTSS can monitor these kinds of motions. The PTSS can be applied to body joint surfaces easily to monitor the subtle changes of athletes. It can meet the requirements of many sports events with difficult skills monitoring. In addition, it can capture the motion signals of diving athletes and transmit them without a power supply in real-time. Meanwhile, for monitoring the violent motions of athletes, the PTSS can be combined with two transparent and stretchable hydrogel electrodes. The electrodes only conduct electricity, but also have excellent stretchability. Figure 1a shows a scene of athletes wearing a PTSS to monitor the motions and sense of signal transmission. In this process, the athletes wear PTSS to monitor angle and frequency changes. Then, the sensing signal generated by the PTSS is transmitted to other electrical equipment. At the same time, the PTSS can convert mechanical energy into electrical energy, which provides more possibilities for carbon neutralization and peak emission of carbon dioxide in the sports monitoring field. Figure 1b shows the optical image of PTSS. The PTSS (I) is made up of TENG (II), PENG (III), and hydrogel electrodes (IV). This sensor has the characteristics of high transparency and high stretchability. The characteristic of high transparency can ensure that the coach can observe detailed motions in order to analyze athletes’ motions by combining the sensing signals. It is noted that excellent stretchability is the core of this work. In order to cope with strenuous exercise, we use a soft and stretchable hydrogel electrode to represent the traditional rigid electrode. It can conduct electricity and respond to enormous deformation, ensuring the smooth transmission of sensing signals. Figure 1c shows the manufacturing steps of the hydrogel. AM, MBA, APS, and TMED are mixed in a certain proportion to form a hydrogel electrode. Figure 1d shows the manufacturing steps of the PTSS. The PTSS is composed of PENG and TENG. Step I shows the manufacturing steps of PENG. In this process, PVDF powder is manufactured into PVDF film. Then, the PVDF film is covered with the anti-short circuit treatment. Finally, the double hydrogel electrodes are fixed on the PVDF by the PTFE. Step II shows the manufacturing steps of the PDMS. The PDMS solution and curing agent are mixed according to the weight ratio of 10:1, then bubbles are removed, and the mixed solution is heated and molded. Then, the PDMS film is cut into a hollow structure and attached to PENG so that the PTFE substrate of PENG can rub against the skin.

**Figure 2:**
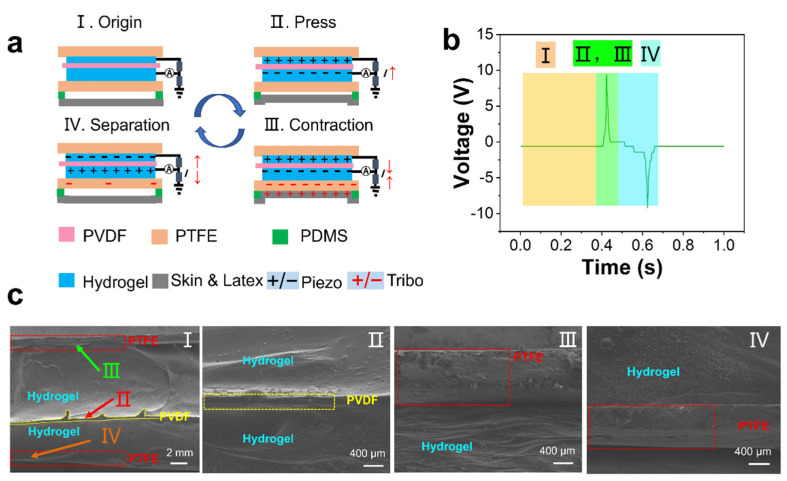
The mechanism of PTSS and SEM of PENG. (**a**) Coupling mechanism of TENG and PENG. (**b**) Corresponding signal. (**c**) The SEM image of PENG.

Figure 2 shows the coupling mechanism of TENG and PENG. TENG and PENG use a common electrode. The TENG system adopts a single electrode mode. The coupling mechanism can be divided into four sections. When the athlete is in a static state, the PTSS is in a free state with no external force and no electrical output (State I and Figure 2(bI)). When an external force is applied to the PTSS, the deformation happens on the PENG firstly, and the piezoelectric signal is produced (State II and Figure 2(bII)) with the external force increasing continuously. The skin/latex contacts the PTFE, the skin/latex tends to lose electrons, while the PTFE tends to acquire electrons, electrons transfer from the skin to the PTFE. The negative side of the PVDF and negative layer of TENG share the same electrode. The contact electrification and piezoelectricity occur simultaneously. Therefore, the electrons of the PENG and TENG move in the opposite direction on the circuit (State III and Figure 2(bIII)). When the external force disappears, the electrons flow back. The electrons of the PTFE flow to the ground. Then, the electrons of PENG and TENG move on the circuit in opposite directions again (State IV and Figure 2(bIV)). This unique working mechanism can be well applied to sports. Many sports are periodic, thus, many motion structures can be directly coupled with this mechanism in sports. Therefore, PTSS can be applied to human motion monitoring perfectly. Figure 2c shows the SEM (HITACHI S-4800) image of PENG. The Figure 2(cI) shows the side-view SEM image of the whole device. The PTSS is packaged by the PTFE film. Figure 2(cII–cIV) are the enlarged view of Figure 2(cI). There is no gap between the hydrogel and the PVDF layer. Hence, the PENG unit can hardly be influenced by the triboelectric signal.

**Figure 3:**
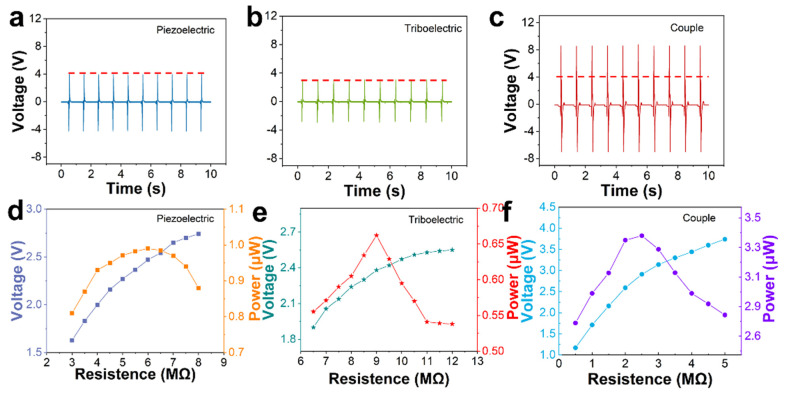
Electrical properties of PENG, TENG, and PTSS in a 100% RH environment. (**a**–**c**) Comparison of output voltages of PENG, TENG and PTSS. (**d**–**f**) Power and outputting voltage of PENG, TENG, and PTSS at different load resistances.

In this study, the outputting voltage is the major factor in the monitoring process. We injected water mist into the airtight box and the humidity achieved 100%. Figure S1 shows the cross-sectional microscopic image of the PTSS and three views of the device. It can be clearly seen that there is no air gap in the PENG unit, which can avoid the influence of the triboelectric effect between the hydrogel and the PVDF. The device is packaged by Dow Corning 3140 RTV. Therefore, the hydrogel cannot dehydrate in a short time, as this improves the stability and extends its service life. A stepper motor was used to test the outputting voltage of PENG, TENG, and PTSS at the 100% RH environment (Figure S2) and air, respectively. The average outputting voltage of PENG, TENG, and PTSS was 3.96 V, 3.08 V, and 8.6 V, respectively in Figure 3a–c. Figure S3 shows the data measured by PENG, TENG, and PTSS under the air condition and tested force, no deformation happened to them. We applied a tiny force to them; the applied force was 15 N. It is evident that PENG and TENG can be coupled together. Compared with PENG and TENG, the PTSS has an excellent sensing performance. Some movements cannot be detected by a single device. TENG cannot monitor the twist movement clearly. The outputting and response of PENG are low. The PTSS combines the merits of TENG and PENG. It can ensure a high output and accurate detection under complicated conditions such as twisting and bending. In addition, PTSS can increase the range of movement monitoring. We tested the 360° wrist rotation by PENG, TENG and PTSS in the Figure S4. Compared to single PENG and TENG, the PTSS can monitor more detailed signals. It is a device that can monitor various movement states. It can help the coach to better analyze the signal. The coach can observe clearer signals in the monitoring process. Figure 3d–f shows the power and outputting voltage of the PENG unit, the TENG unit, and the PTSS at different load resistances. As the resistance increases, the outputting voltage also increases. When the load resistances are 2.5 MΩ, 6 MΩ, and 9 MΩ, the power of PTSS, TENG, and PENG reaches the maximum. Therefore, the inherent resistance of PTSS, TENG, and PENG are 2 MΩ, 6 MΩ, and 9 MΩ, respectively.

**Figure 4:**
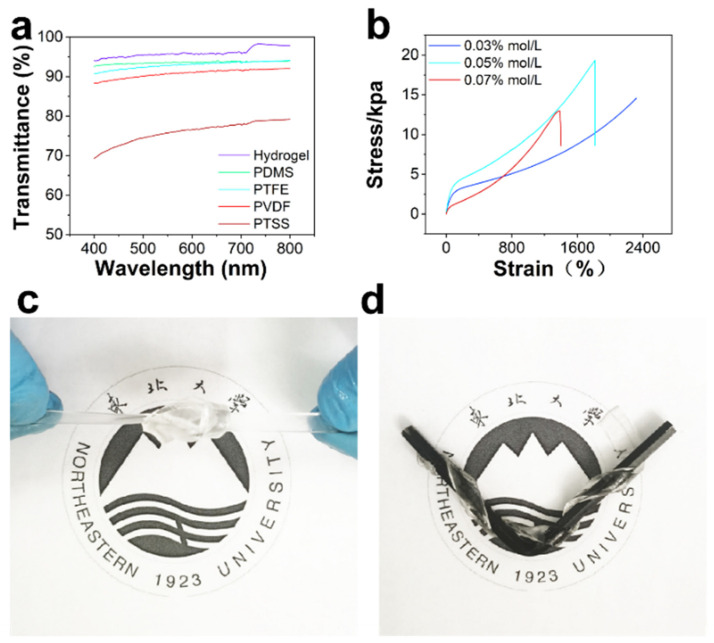
Physical properties of hydrogel (**a**) Transparency of each part of the PTSS. (**b**) Stress-strain curve of different APS ratios of hydrogel. (**c**,**d**) Scene of stretching hydrogel in any shapes.

Hydrogel is used as an electrode; hydrogel has excellent characteristics and can be well used for motion monitoring. We tested the resistance of hydrogel and found that with the stretching of hydrogel, its resistance increases. When the hydrogel is stretched from 2 cm to 12 cm, the resistance changes from 54.5 kΩ to 96.8 kΩ (Figure S5). Even if the resistance of the hydrogel changes with stretching, the inherent resistance of the PTSS is much larger than that of the hydrogel. Therefore, the change in hydrogel resistance does not affect the outputting voltage. Compared with a metal electrode, the hydrogel electrode has a similar sensing signal. The hydrogel has good stretchability, biocompatibility, and comfort properties and these are better than those of the metal electrode (Figure S5). Figure 4a shows the transparency of the PTSS and each part of it. The average transmittance of hydrogel for the PDMS, PTFE, PVDF, and PTSS are 96.06%, 93.55%, 92.92%, 90.79%, and 76%, respectively. The above data demonstrate that the PTSS has high transmittance, thus a coach can observe detailed motions conveniently. Figure 4b shows the stress-strain curve of different APS ratios of the hydrogel. We found that although the proportion of APS is very small, the content of APS is an important index that affects hydrogel stretchability and solidification time. The stress-strain curves were tested by changing the APS content to 0.01 mol/L, 0.03 mol/L, 0.05 mol/L, and 0.07 mol/L, respectively, while the concentration of other materials remained constant. We found that the hydrogel with a concentration of 0.01 mol/L APS cannot be cured (Figure S6). According to the results, the 0.03 mol/L APS hydrogel has the best tensile property which can be stretched to 2317.53% at least. This is because the maximum range of the machine is 2317.53%. The 0.05 mol/L and 0.07 mol/L APS hydrogel are 1815.21% and 1400.93%, respectively. The optical images of the tensile property are shown in Figure S7. The excellent tensile properties can prevent the electrode from being damaged. It ensures the continuous operation of the motion monitor. Figure 4c,d show that hydrogels can be stretched in many structures. In sport monitoring, in order to meet the needs of sports, hydrogels can be equipped with different structures to reduce the impact on athletes.

**Figure 5:**
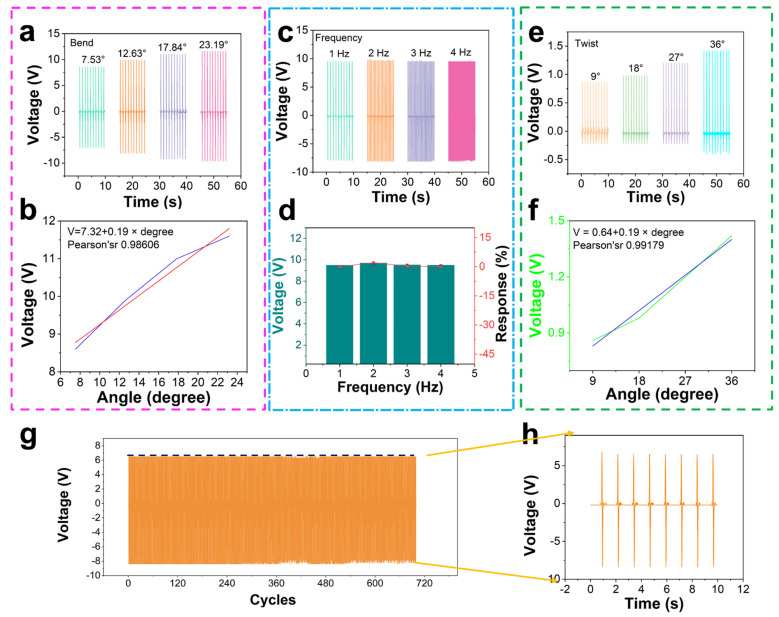
The performance test of PTSS. (**a**) Outputting voltage of PTSS at different bend angles in 100% RH. (**b**) The linear relation of angles and voltages. (**c**,**d**) Outputting voltage and response of PTSS at different frequencies in 100% RH. (**e**) Outputting voltage of PTSS at different twist angles in 100% RH. (**f**) Linear relation of twist angles and voltages. (**g**) Durability test of PTSS. (**h**) The detail of durability.

As a motion monitoring sensor, its sensing accuracy and size, including bend angle, frequency, and twist angle should be taken into account. Usually, water sports are monitored under high humidity conditions. We did the above experiments in 100% RH (Figure S2), all of which were tested using a stepping motor. Figure 5a shows the PTSS outputting voltage at different bending angles. When the bend angle is 7.53°, 12.63°, 17.84° and 23.19°, the outputting voltage of PTSS in 100% RH is 8.6 V, 9.9 V, 11 V, and 11.6 V. The data measured under air condition is shown in Figure S8. Figure 5b shows the linear relationship between angles and voltages of the PTSS in the 100% RH environment. The Pearson coefficient is 0.98606, which indicates that it has a good linear relationship. The formula is:

*V* ≈ 7.32 + 0.19 × degree (1)

To study the relation of bend angle, frequency, and twist angel with output voltage. The response of the PTSS can be calculated from the following equation:

(2) $$R\%=\left|\frac{V_{0}-V_{i}}{V_{i}}\right|\times 100\%,$$

where V_0_ and V_i_ are the outputting voltage of 7.53° (first data) and other angles. When the bend angle is 7.53°, 2.63°, 7.84°, and 23.19°, the response of the PTSS is 0, 13.13%, 21.82%, and 25.86% (Figure S9). Figure 5c shows the outputting voltage of the PTSS at the same angle and at different frequencies. When the frequency is 1 Hz, 2 Hz, 3 Hz, and 4 Hz, the outputting voltage is 9.5 V, 9.7 V, 9.52 V, and 9.51 V. The data measured under air condition has been shown in Figure S8. Figure 5d shows the responses of the PTSS at the same angle and different frequencies. The two dielectric plates, with thicknesses of d1 and d2 and the relative dielectric constants ε_r1_ and ε_r2_, respectively, are stacked face to face as two triboelectric layers. At the outer surface of the PTFE dielectric, a hydrogel layer is deposited as an electrode. The distance (*x*) between the two triboelectric layers can be varied under the agitation of mechanical force. After being forced to get in contact with each other, the inner surfaces of the two triboelectric layers will have opposite static charges (tribo-charges) with equal density of σ, as a result of contact electrification. For insulators, as discussed, it is reasonable to assume that the tribo-charges are uniformly distributed along the two surfaces with negligible decay. When the two triboelectric layers start to separate from each other, with increased *x*, a potential difference (*V*) between the two electrodes will be induced. The amount of transferred charges at the electrode, as driven by the induced potential, is defined as *Q* which also represents the instantaneous amount of charges on the electrode. With the above model, the *V*–*Q*–*x* relationship of such contact-mode TENG can be derived based on electrodynamics. Since the area size (*S*) of the PTFE and skin is several orders of magnitude larger than their separation distance (*d*_1_ + *d*_2_ + *x*) in the experimental case, it is reasonable to assume that the two electrodes are infinitely large. Under this assumption, the charges will uniformly distribute on the inner surfaces of the PTFE and skin. Inside the dielectrics and the air gap, the electric field only has the component in the direction perpendicular to the surface, with the positive value pointing to hydrogel. From the Gauss theorem, the electric field strength at each region is given by:

(3) $$\mathrm{Inside}\;\mathrm{Dielectric}\;\mathrm{skin}:\;E_{1}=-\frac{Q}{S_{\varepsilon_{0}\varepsilon_{r1}}}$$

(4) $$\mathrm{Inside}\;\mathrm{the}\;\mathrm{air}\;\mathrm{gap}:\;E_{air}=\frac{-\frac{Q}{S}+\sigma \left(t\right)}{\varepsilon_{0}}$$

(5) $$\mathrm{Inside}\;\mathrm{Dielectric}\;\mathrm{PTFE}{:}_{\;}E_{2}=-\frac{Q}{S_{\varepsilon_{0}\varepsilon_{r2}}}$$

The voltage between the two electrodes can be given by:

(6) $$V=E_{1}d_{1}+E_{2}d_{2}+E_{air}x$$

Therefore, the outputting voltage is dependent on the surface charge density and the motion frequency depends on the voltage frequency [48,49]. When the frequency is 1 Hz, 2 Hz, 3 Hz, and 4 Hz, the response is 0, 2%, 0.2%, and 0.05%, respectively (Movie S1). The response demonstrates that when the bend angle is fixed, the motion frequency changes and the outputting voltage is stable. The frequency of the voltage peak occurrence is the same as that of motion. Therefore, PTSS can monitor the motion frequency with excellent performance. For example, in the short race, step frequency and step length are the absolute factors of the competition. Monitoring the step frequency at the starting stage, running stage, and sprint stage is a vital measure of an athlete’s performance. The PTSS can monitor the frequency of every stage to provide visual data. Figure 5e shows the outputting voltage of the PTSS at different twist angles. When the twist angles are 9°, 18°, 27°, and 36°, the average voltages of the PTSS are 0.86 V, 0.98 V, 1.2 V, and 1.42 V, respectively. The data measured under air condition is shown in Figure S8. Figure 5f shows the linear relation of twist angle and voltage. The Pearson coefficient is 0.99179, which demonstrates that there is a good linear relationship. The formula is:

*V* ≈ 0.64 + 0.19 × degree (7)

When the twisted angel is 9°, 18°, 27°, and 36°, the response is 0, 12.24%, 28.33%, and 39.43% (Figure S9). Figure 5g shows the durability test for PTSS. PTSS still has excellent outputting performance after working at a big bend angle for 720 cycles. It demonstrates that PTSS can work in violent motions. Meanwhile, we pressed the hydrogel for 3400 cycles, and it also had an excellent working performance (Movie S2). Figure 5h shows the details of durability. It shows that the device can maintain a stable sensing characteristic.

**Figure 6:**
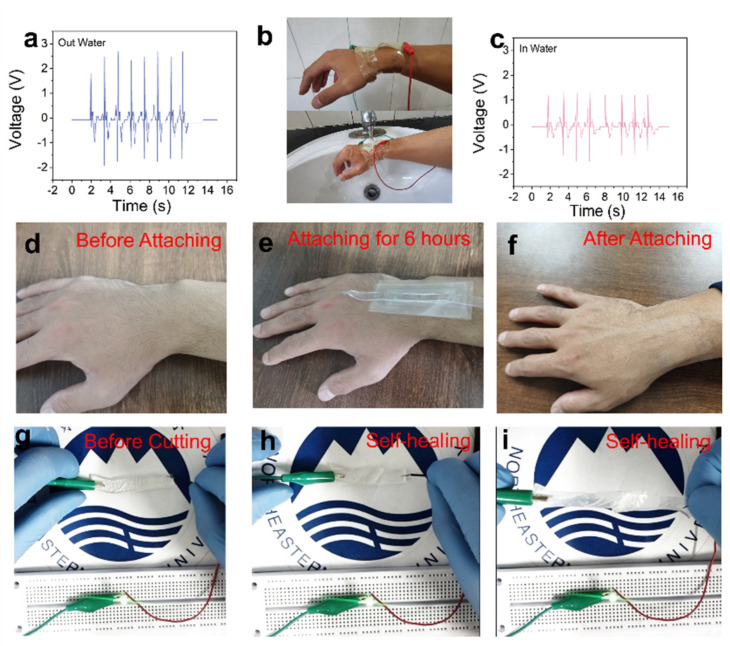
Scenes of waterproof, biocompatibility, and self-healing. (**a**–**c**) Test of waterproof ability. (**d**–**f**) Test of biocompatibility. (**g**–**i**) Self-healing property of hydrogel.

To meet the extreme monitoring conditions, we tested the performance of PTSS in the water (Movie S3). Figure 6a shows the outputting voltage of the wrist bending before entering the water. Before entering the water (Figure 6b), the PENG and TENG of the PTSS worked together, with an average voltage of 2.45 V, after entering the water, the outputting voltage dropped, with an average voltage of 1.25 V (Figure 6c). This is because the friction layer of the PTSS directly rubs against the skin, and the water makes electrons flow to the ground with the water. Therefore, TENG does not work at this time. However, the PENG is tightly fixed by the PTFE. The PENG has the excellent property of being waterproof. Figure 6d–f shows the biocompatibility of the PTSS. The PTSS was attached to the skin for 6 h, and there was no rejection reaction on the athlete’s wrist. This demonstrates that PTSS has excellent biocompatibility and it can monitor the motion of athletes for a long time. Figure 6g–i shows the self-healing property of hydrogel. In the violent motion, the electrode may be damaged. The hydrogel can meet this problem because it has a good tensile ability and self-healing properties. Figure 6g shows that hydrogel can be used as a wire way to light the LED, and it has good stability and self-healing properties. After the hydrogel is cut, it can also be connected to conduct electricity (Figure 6h). At the same time, it also has a good stretching ability to the motion display (Movie S4), and hydrogels have self-healing properties and can be stretched 70.11% after repair (Figure S10). Due to the self-healing function of hydrogel, the hydrogel electrode can recover by itself even though the damage occurs at a high strength impact. It improves the stability and extends the service life [50]. It is noted that stretchability like this cannot meet the intense exercise demand, but it can be applied to many static motions such as weightlifting.

**Figure 7:**
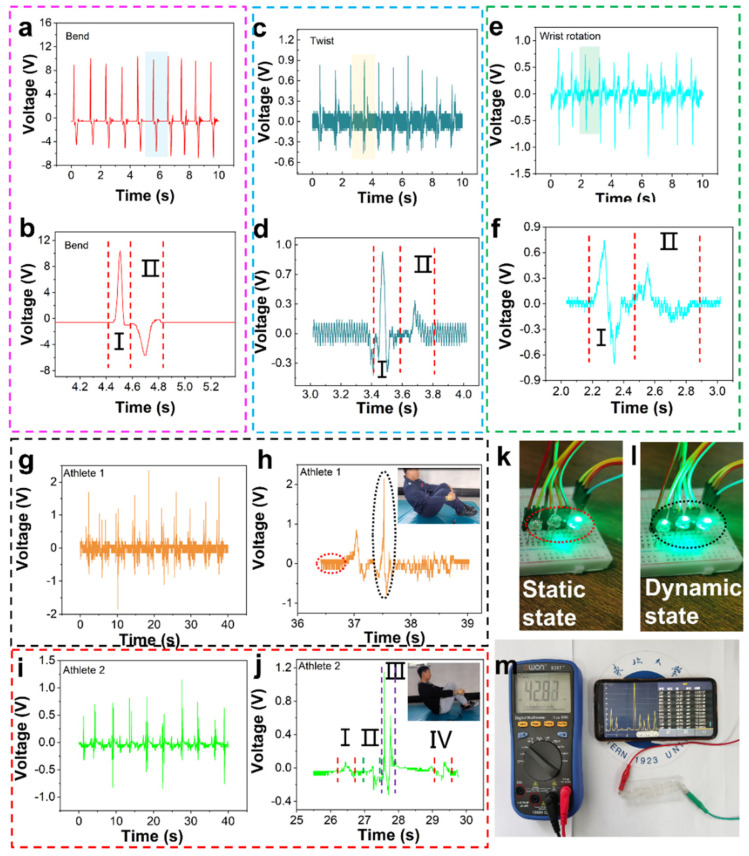
The actual test and wireless signal transmission system. (**a**,**b**) Wrist bending test and its response. (**c**,**d**) Wrist twist test and its response. (**e**,**f**) Wrist rotation test and its response. (**g**,**h**) Outputting voltage and details of Athlete 1′s 301C diving motion. (**i**,**j**) Outputting voltage and details of Athlete 2′s 301c diving motion. (**k**–**m**) Wireless Bluetooth transmission system.

The technical motions of diving are difficult and highly ornamental. The referees score for the athletes’ performance, including their handstands or upright preparation postures, aerial skills, the number of somersaults, and the spray size at the moment of entering the water. At present, the water rip entry motion of the world’s top athletes is to cross their hands to form a square shape, and the water spray formed by this technical motion is small. In order to better monitor the athletes’ technique of rip entry motion, we must monitor their wrist motions firstly, which are wrist bending, twisting, and rotation. The voltage generated by a series of motions is shown in Figure 7a,b. Figure 7a,b shows the outputting voltage and details of wrist bending motion, and its specific motions are shown in Movie S5. The bending of the athlete’s wrist produces an upper wave peak I, and the straightening of the athlete’s wrist produces a lower wave peak II. The reason why wave peak I is significantly greater than wave peak II is that the speed and strength of the bending motion are greater than those of the straightening motion. This kind of motion monitoring is also suitable for shotput wrist motion monitoring. Figure 7c,d shows twist motions and details of the twist, its specific motions are shown in Movie S6. The rotation motion of the wrist includes internal rotation and external rotation. In Figure 7d, part I is the internal rotation motion and part II is the external rotation motion. As shown in Figure 7d, wave peak I is significantly larger than wave peak II because the speed and strength of the internal rotation motion are greater than those of the external rotation motion. This kind of motion monitoring is also applicable to the wrist-twisting and pulling motion monitoring of table tennis. We believe that it will be an important research direction to apply this monitoring method to the stability monitoring of athletes’ rotation technical motion. Figure 7e,f shows the rotation motion and details of the wrist. Part I of Figure 7f is the internal rotation motion and part II is the external rotation motion. Wave peak I is significantly greater than wave peak II, because the speed and strength of internal rotation motion are greater than those of external rotation motion (Movie S7). This kind of motion monitoring is also applicable to the wrist motion monitoring of diving athletes. Based on the limitations of this research, our group cannot quantitatively analyze its speed and strength, which is also a direction for our next study. Figure 7g–j show the motions and details of two athletes simulating 301C diving on land (Movies S8 and S9). Figure 7h,j show signal waveforms of two athletes in one motion cycle. Overall, the waveforms of the two athletes are similar, but there are also some differences. Figure 7j shows a 301C motion with a knee hugging reverse somersault for half a cycle and a difficulty coefficient of 1.8. 301C motion is divided into four stages. Stage I is the round body motion of holding the knees with both hands, and a certain angle of the wrist. As shown in Figure 7j, Athlete 2 has a large and complete knee hugging motion in stage I. Stage II is a tight state in which the arms are quickly extended from the knee hugging state to both sides of the body. At this time, the wrist experiences bending from holding the knee to straightening. Stage III is the water entry stage. The arms quickly move from both sides of the body to the top of the head, and the hands form a square shape. At this time, the wrist needs to rotate inward from the straight state to the square state. After entering the water, the wrist naturally returns to the normal state. The operation signal at this time and Figure 7f are the voltage generated by the same operation. Stage IV is the stage of re-embracing the knee, and the motion is the same as that in stage I. In addition, we have also monitored the twisting and pulling motion of table tennis (Movie S10), and the sensing signal is shown in Figure S11. It shows that PTSS can monitor multiple motions. Figure 7k,l are a schematic diagram of the wireless Bluetooth transmission system. The sensor is connected to a signal transmitting module. When the transmitting module receives the signal, it transmits the signal to the receiving module, and the LEDs are controlled by it. When the athlete is in a static state, there is no signal and Bluetooth only lights up one light, corresponding to the red circle state in Figure 7h. In contrast, when the athlete is in a dynamic state, the signal is produced and Bluetooth lights up three lights, corresponding to the black circle state in Figure 7h. The encapsulated device connects with the Bluetooth module. In the Bluetooth communication test, we only put the PTSS into the water. In this study, the main aim was to design a sensor which can be used to monitor the human movement underwater. The experiment showed that PTSS can monitor the bending and twist signal underwater. We have encapsulated the device and Bluetooth together. It will be introduced in subsequent work. Figure 7m shows the real-time wireless Bluetooth waveform display system (Movie S11). In the process of training, if we can collect information through mobile phones, it will be conducive to more direct monitoring of athletes’ training status. Therefore, we built a wireless monitoring system composed of a hybrid nano-generator, digital multimeter with Bluetooth module and collection to demonstrate the possibility of human motion monitoring (Figure 7m). Wearable mixed-mode sensors can collect and process human motion data through a digital multimeter, and a mobile app can monitor the voltage in real-time. We have proved that it can be applied to human motion monitoring through a simple impact test. Meanwhile, it can also charge capacitor. Figure S12 shows curves of the PTSS charges capacitors. The PTSS charges are 0.1 μF, 0.22 μF, and 0.47 μF and the charging voltages are 2.05 V, 1.79 V and 0.86 V, respectively. The PTSS can not only generate electricity by itself, but can also convert mechanical energy into electrical energy. This is of great significance to carbon neutralization and peak carbon dioxide emissions. This potential application provides more possibilities for the field of motion detection.

## 4. Conclusions

To conclude, a new type of flexible stretchable self-healing composite nano-generator for human motion monitoring sensor has been developed. It is composed of PENG, TENG, and hydrogel electrodes. The contact-separation mode is adopted to realize the coupling of the piezoelectric effect and triboelectric effects, which further improve the sensitivity and measuring range. The hydrogel electrode has excellent stretchability and has a self-powered property which can meet the requirement of strenuous exercise. At the same time, PTSS can monitor different movements and events. It has a good monitoring effect on difficult and beautiful sports events. This research also solves the problem of wireless transmission and brings more opportunities for wireless big data and scientific sports.

## Data Availability

The data presented in this study are available on request from the corresponding author. The authors would like to thank Jilong Gao from Shiyanjia Lab (www.shiyanjia.com) for the Stress and strain analysis.

## Supplementary Materials

The following are available online at https://www.mdpi.com/article/10.3390/nano12010104/s1; Figure S1: (a) The cross-sectional microscopic image of PTSS (b–d) Three views of the device; Figure S2: The water mist airtight box; Figure S3: (a–c) The outputting voltage of PENG, TENG and PTSS in air test (d) Test force; Figure S4: The outputting voltage of 360° wrist rotation of PENG, TENG, and PTSS; Figure S5: (a,b) The conductivity of hydrogel (c) The outputting voltage of the metal electrode TENG; Figure S6: The image of 0.01 mol/L APS hydrogel; Figure S7: The tensile property of 0.03 mol/L, 0.05 mol/L, and 0.07 mol/L APS hydrogel; Figure S8: The PTSS outputting voltage at different bending angles, frequency, and twist in air; Figure S9: The responses of bending angel and twist angle in 100% RH environment; Figure S10: Stress-strain curve of self-healing hydrogel; Figure S11: The output voltage of twisting and pulling motion of table tennis; Figure S12: Charging characteristic curve; Movie S1: The frequency test of PTSS in 100% RH environment; Movie S2: The hydrogel being pressed for 3400 cycles; Movie S3: The performance of PTSS in water; Movie S4: The self-healing and stretch properties of hydrogel; Movie S5: The wrist bend motion and outputting voltage; Movie S6: The twist motion and outputting voltage; Movie S7: The rotation motion outputting voltage; Movie S8: The motions and details of Athlete 1 simulating 301C diving on land; Movie S9: The motions and details of Athlete 2 simulating 301C diving on land; Movie S10: The twisting and pulling motion of table tennis; Movie S11: The real-time wireless Bluetooth waveform display system.
