# Supplementary material for: A Stretchable and Self-Healing Hybrid Nano-Generator for Human Motion Monitoring
Source: Nanomaterials (Basel). 2021 Dec 29;12(1):104. doi: 10.3390/nano12010104 (PMC8746449; doi:10.3390/nano12010104)
Supplement: Supplementary file 1 [file nanomaterials-12-00104-s001.zip › nanomaterials-1491178/nanomaterials-1491178-Figure , Movie and SI-R3/nanomaterials-1491178-SI-R3.pdf]

A Stretchable and Self-Healing Hybrid Nano-Generator for Human Motion Monitoring

Yongsheng Zhu [1], Fengxin Sun [1], Changjun Jia [1], Tianming Zhao [2,*] and Yupeng Mao [1,*]

[1] Physical Education Department, Northeastern University, Shenyang 110819, China; 2001276@stu.neu.edu.cn (Y.Z.); 2171435@stu.neu.edu.cn (F.S.); 2071367@stu.neu.edu.cn (C.J.)

[2] College of Sciences, Northeastern University, Shenyang 110819, China

* Correspondence: zhaotm@stumail.neu.edu.cn (T.Z.); maoyupeng@pe.neu.edu.cn (Y.M.)

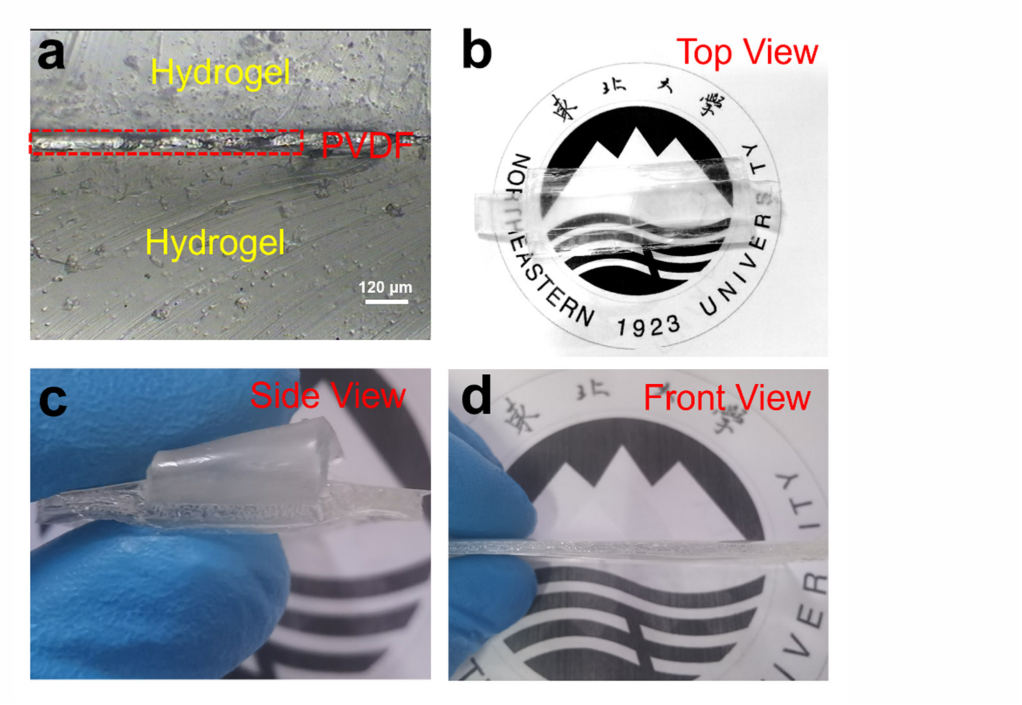

Figure S1. (a) The cross-sectional microscopic image of PTSS (b-c) three views of the device.

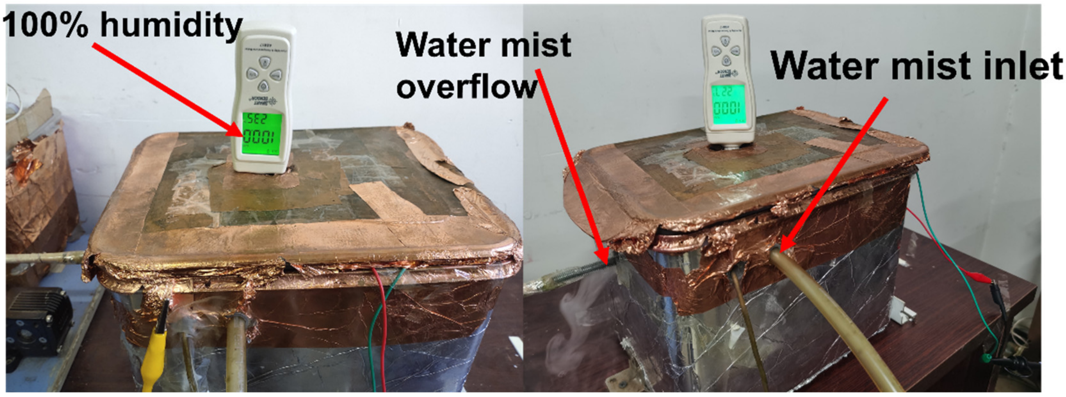

Figure S2. The water mist airtight box.

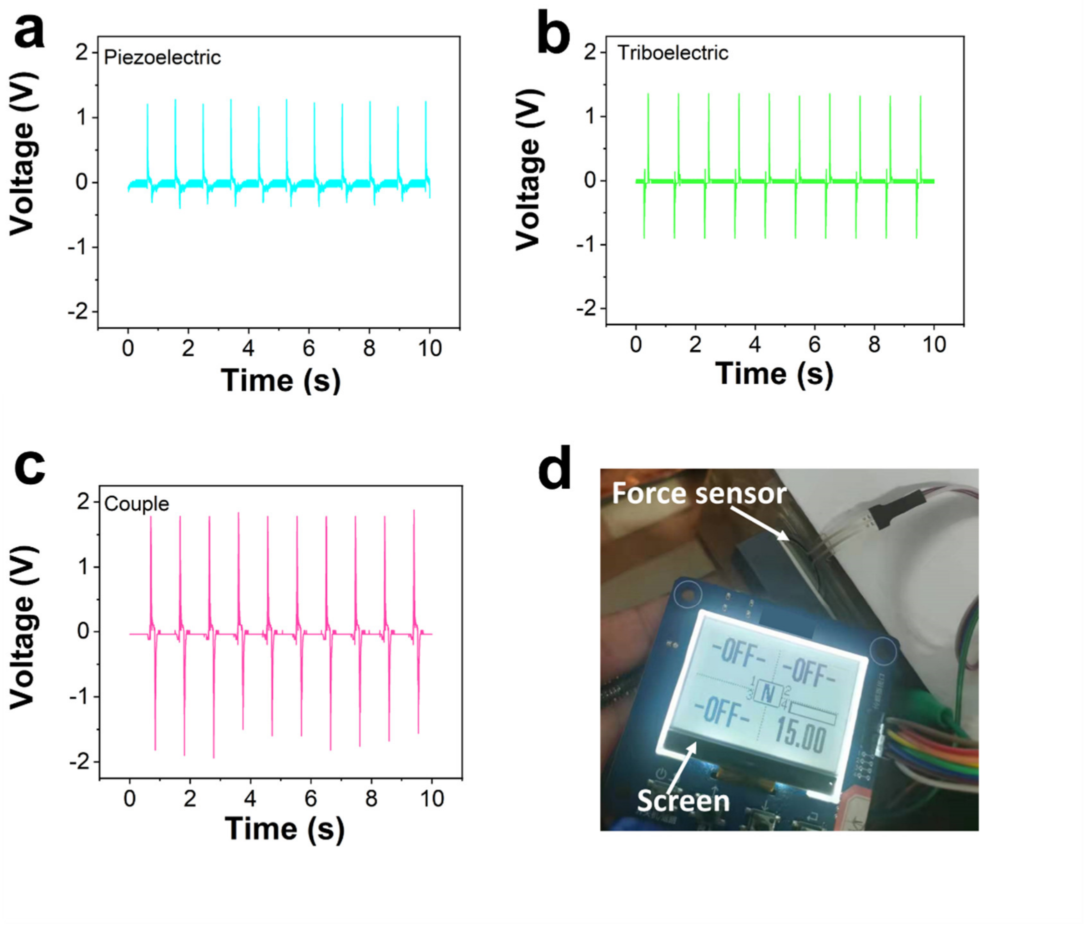

Figure S3. (a-c) The outputting voltage of PENG, TENG of PTSS in air test (d) Test force.

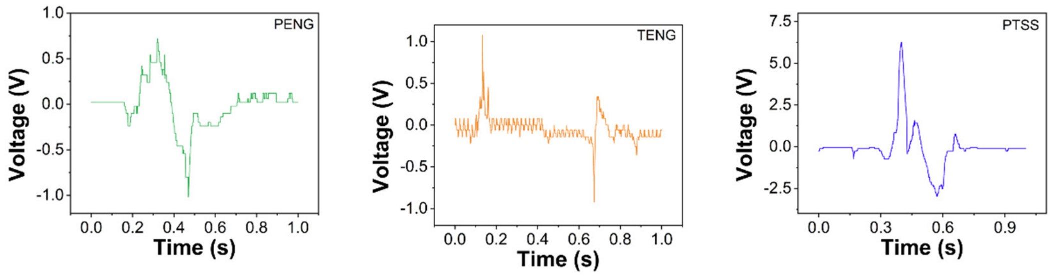

Figure S4. The outputting voltage of 360° wrist rotation of PENG, TENG, and PTSS.

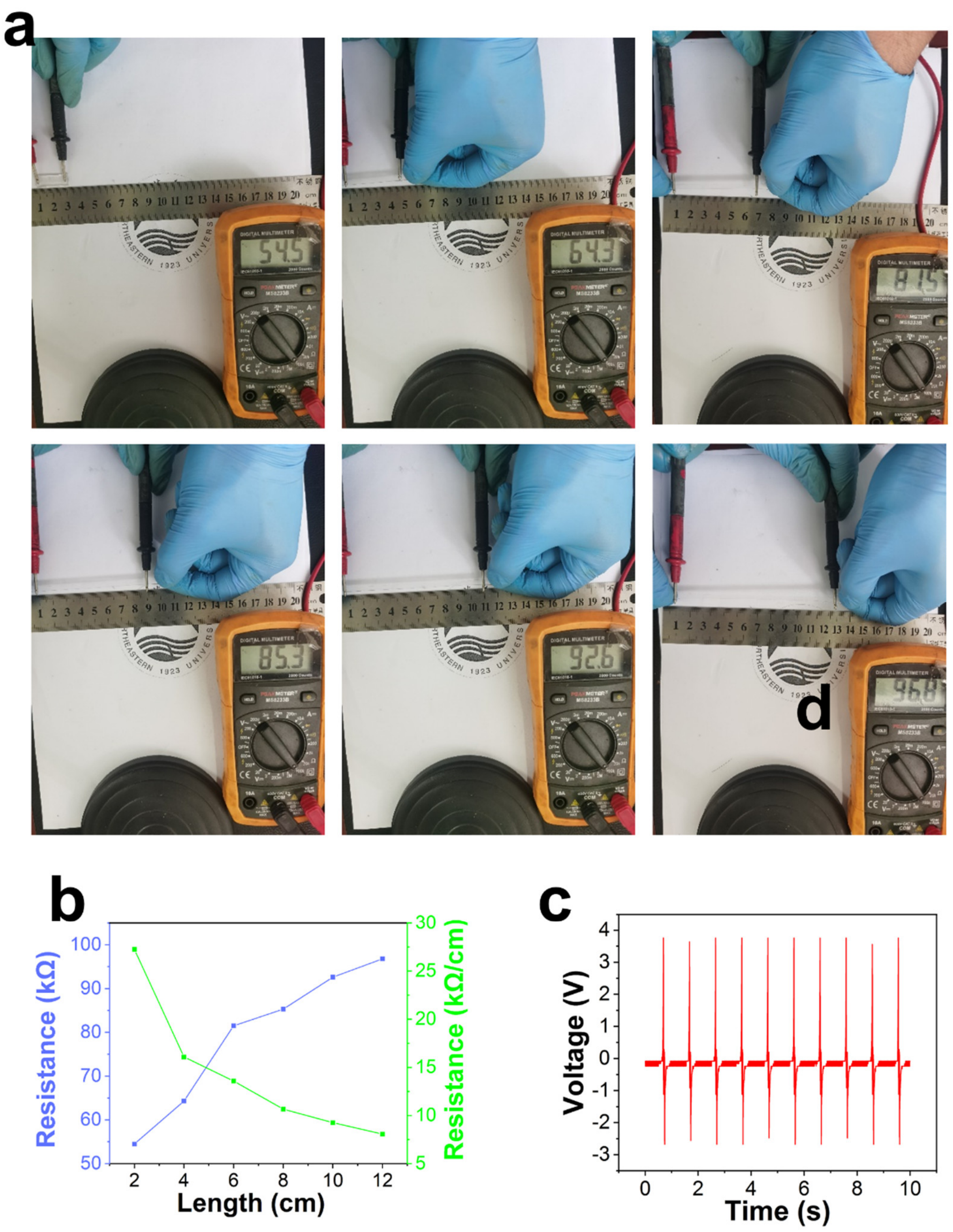

Figure S5. (a-b) The conductivity of hydrogel (c) The outputting voltage of the metal electrode TENG.

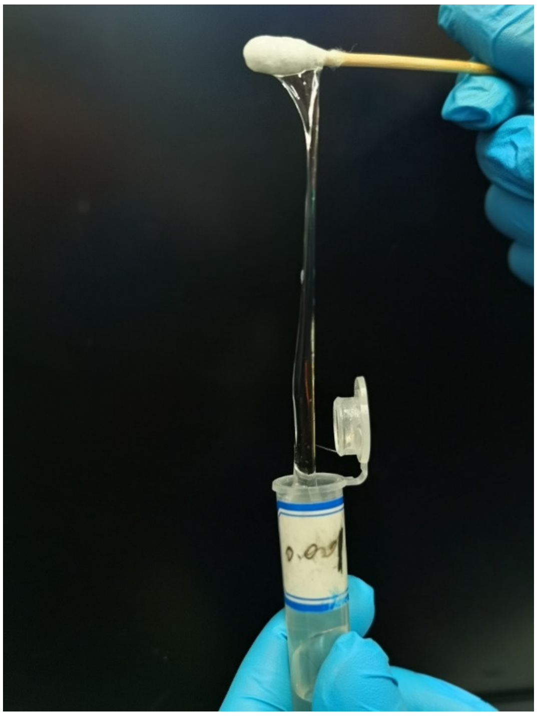

Figure S6. The image of 0.01mol/l APS hydrogel

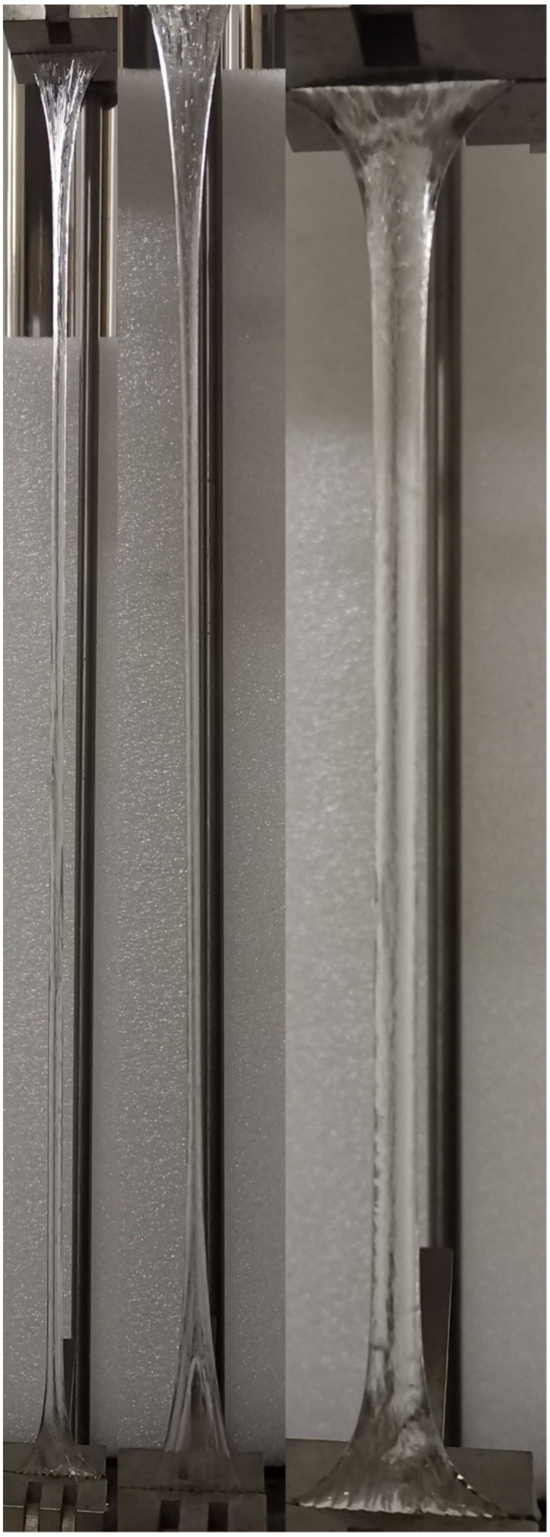

Figure S7. The tensile property of 0.03mol/l, 0.05 mol/l, and 0.07 mol/l APS hydrogel

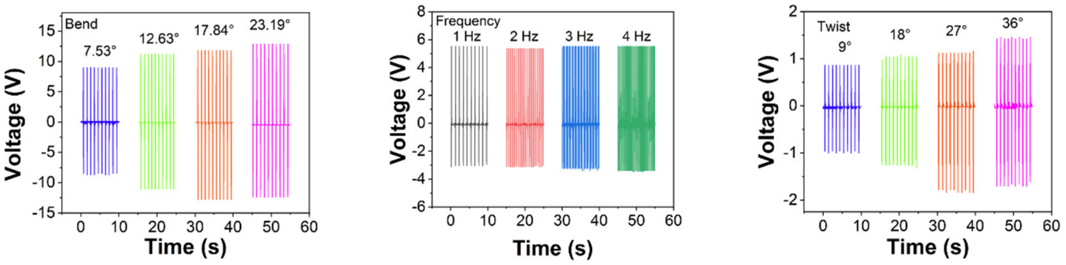

Figure S8. The PTSS outputting voltage at different bending angles, frequency, and twist in ai

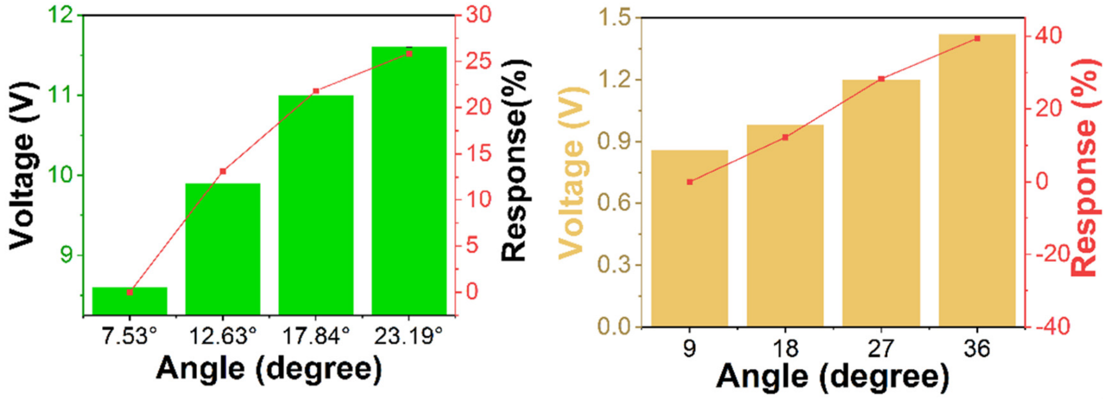

Figure S9. The responses of bend angel and twist angle in 100% RH environment.

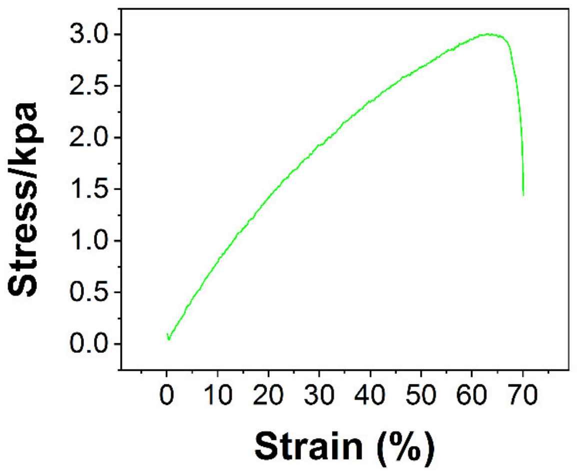

Figure S10. Stress-strain curve of self-healed hydrogel

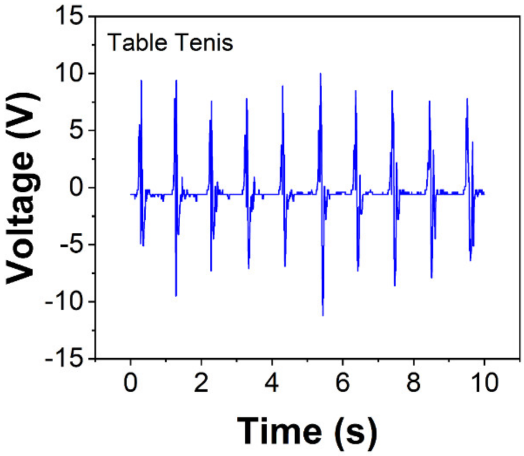

Figure S11. The output voltage of twisting and pulling motion of table tennis.

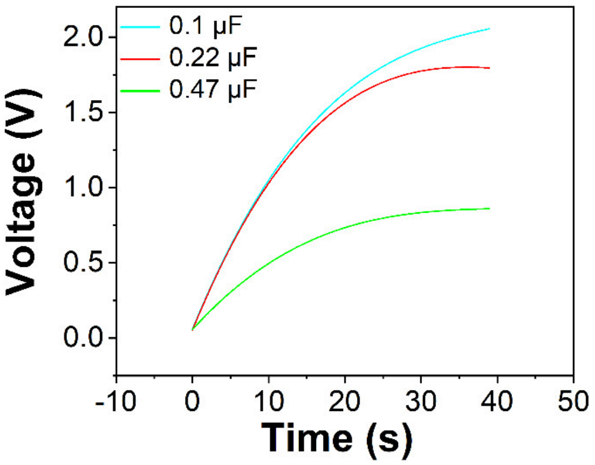

Figure S12. Charging characteristic curve

Supplementary Movie S1. The frequency test of PTSS in 100% RH environment;

Supplementary Movie S2. The hydrogel be pressed for 3400 cycles;

Supplementary Movie S3. The performance of PTSS in the water;

Supplementary Movie S4. The self-healing and stretch properties of hydrogel;

Supplementary Movie S5. The wrist bend motion and outputting voltage;

Supplementary Movie S6. The twist motion and outputting voltage;

Supplementary Movie S7. The rotation motion outputting voltage;

Supplementary Movie S8. The motions and details of athlete 1 simulating 301C diving on land;

Supplementary Movie S9. The motions and details of athlete 2 simulating 301C diving on land;

Supplementary Movie S10. The twisting and pulling motion of table tennis;

Supplementary Movie S11. The real-time wireless Bluetooth waveform display system.
