# Supplementary figures and images for: A Stretchable and Self-Healing Hybrid Nano-Generator for Human Motion Monitoring
Source: Nanomaterials (Basel). 2021 Dec 29;12(1):104. doi: 10.3390/nano12010104 (PMC8746449; doi:10.3390/nano12010104)

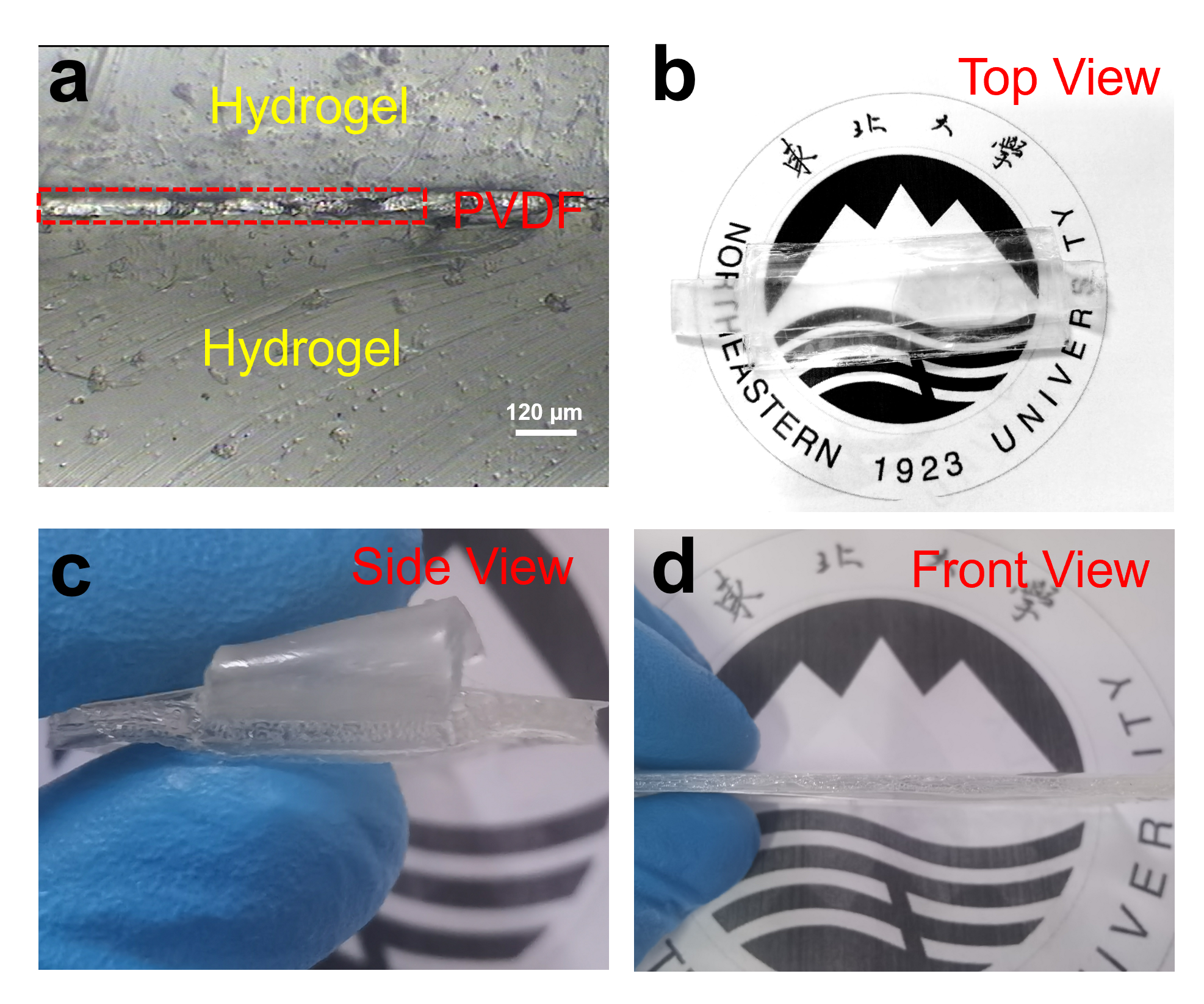

Supplement: Supplementary file 1 [file nanomaterials-12-00104-s001.zip › nanomaterials-1491178/nanomaterials-1491178-Figure , Movie and SI-R3/Fig S1.tif]

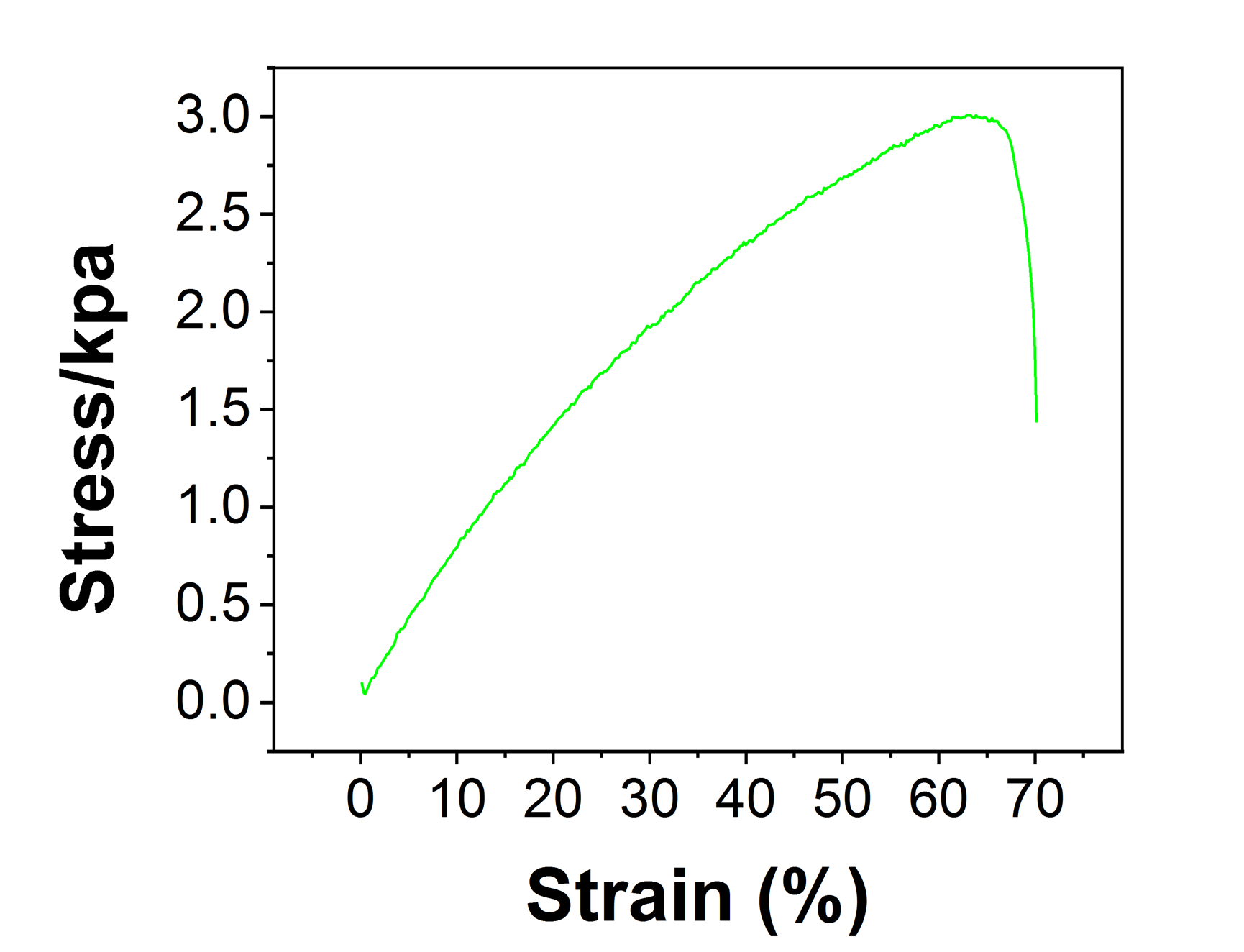

Supplement: Supplementary file 1 [file nanomaterials-12-00104-s001.zip › nanomaterials-1491178/nanomaterials-1491178-Figure , Movie and SI-R3/Fig S10.tif]

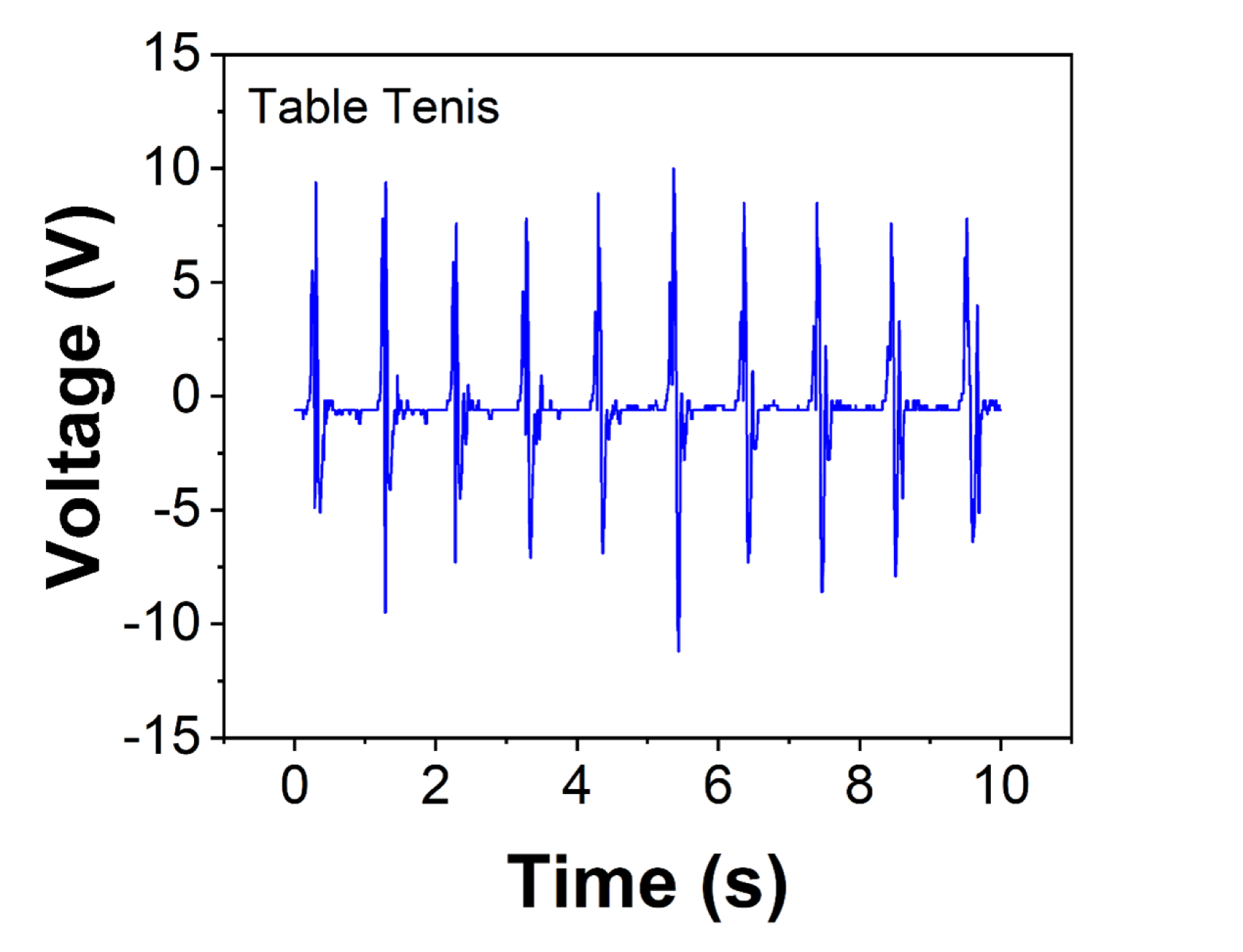

Supplement: Supplementary file 1 [file nanomaterials-12-00104-s001.zip › nanomaterials-1491178/nanomaterials-1491178-Figure , Movie and SI-R3/Fig S11.tif]

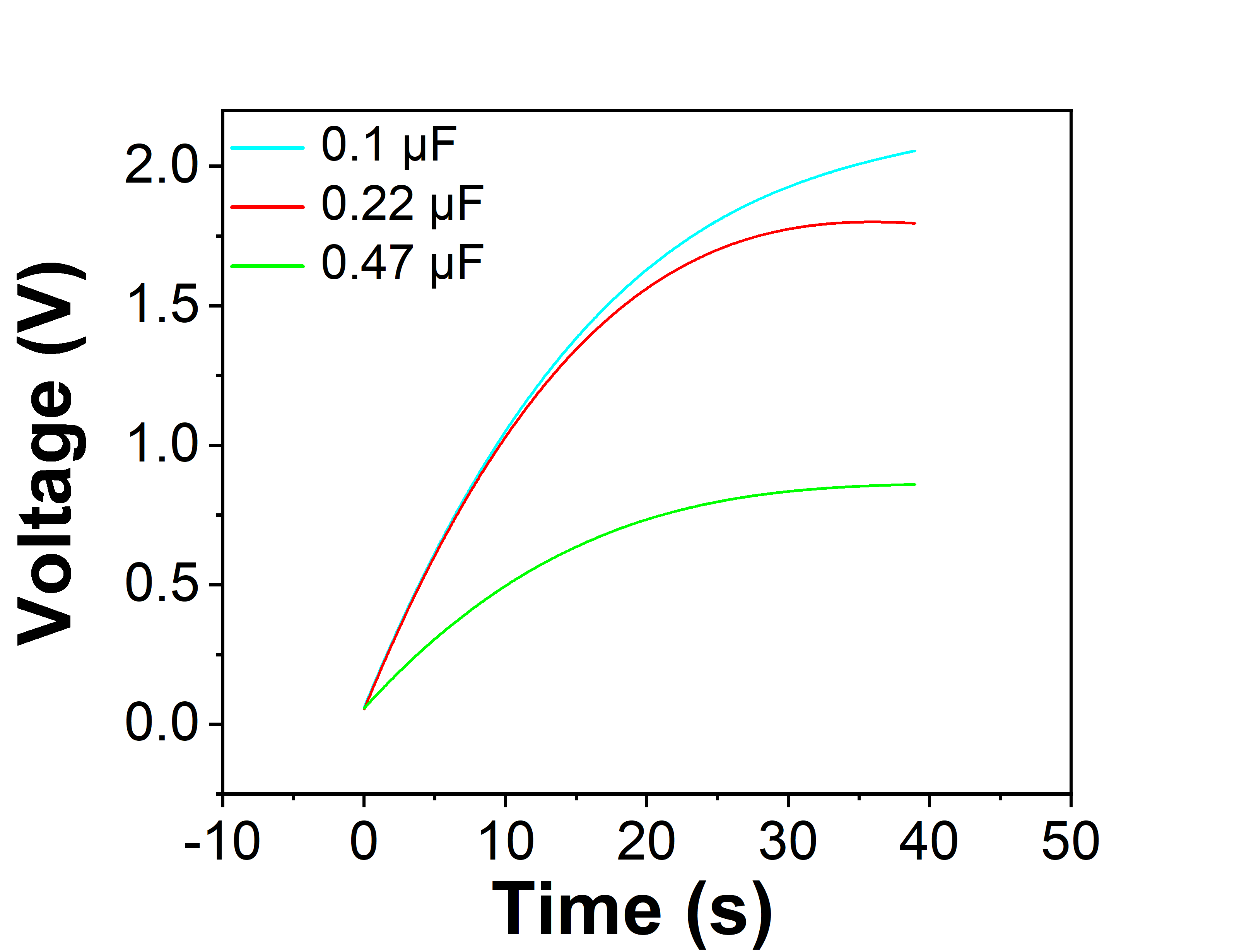

Supplement: Supplementary file 1 [file nanomaterials-12-00104-s001.zip › nanomaterials-1491178/nanomaterials-1491178-Figure , Movie and SI-R3/Fig S12.tif]

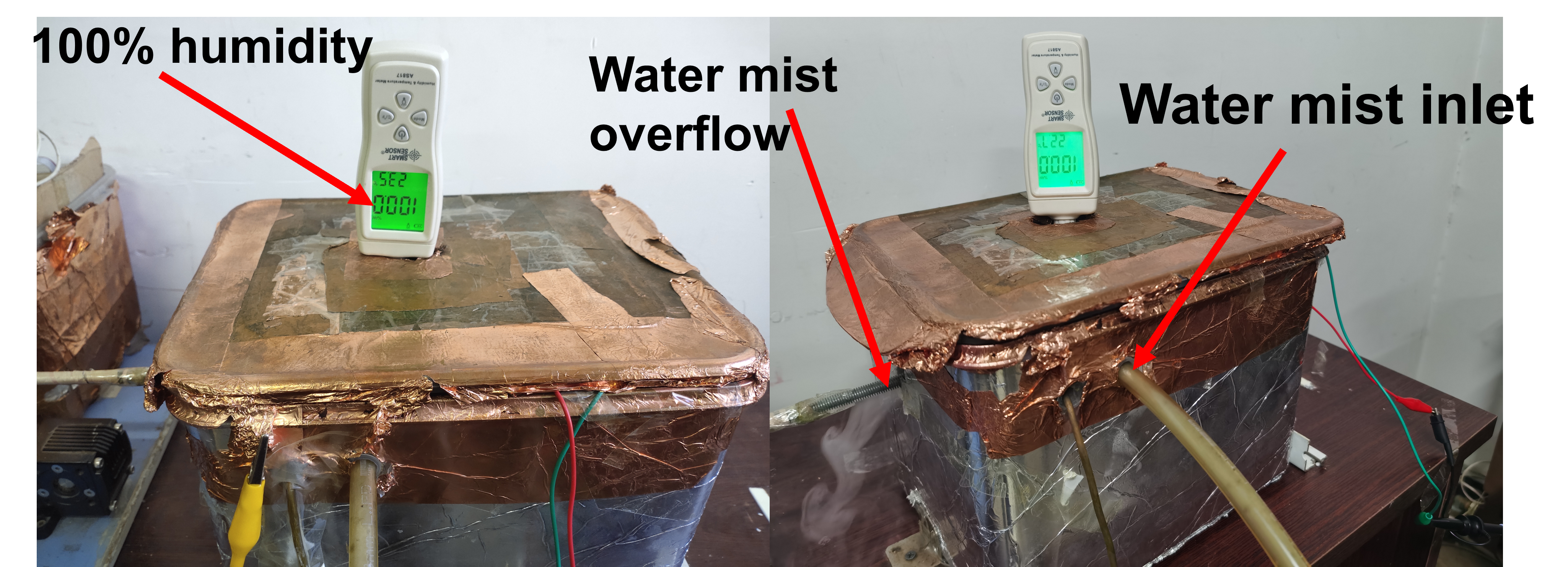

Supplement: Supplementary file 1 [file nanomaterials-12-00104-s001.zip › nanomaterials-1491178/nanomaterials-1491178-Figure , Movie and SI-R3/Fig S2.tif]

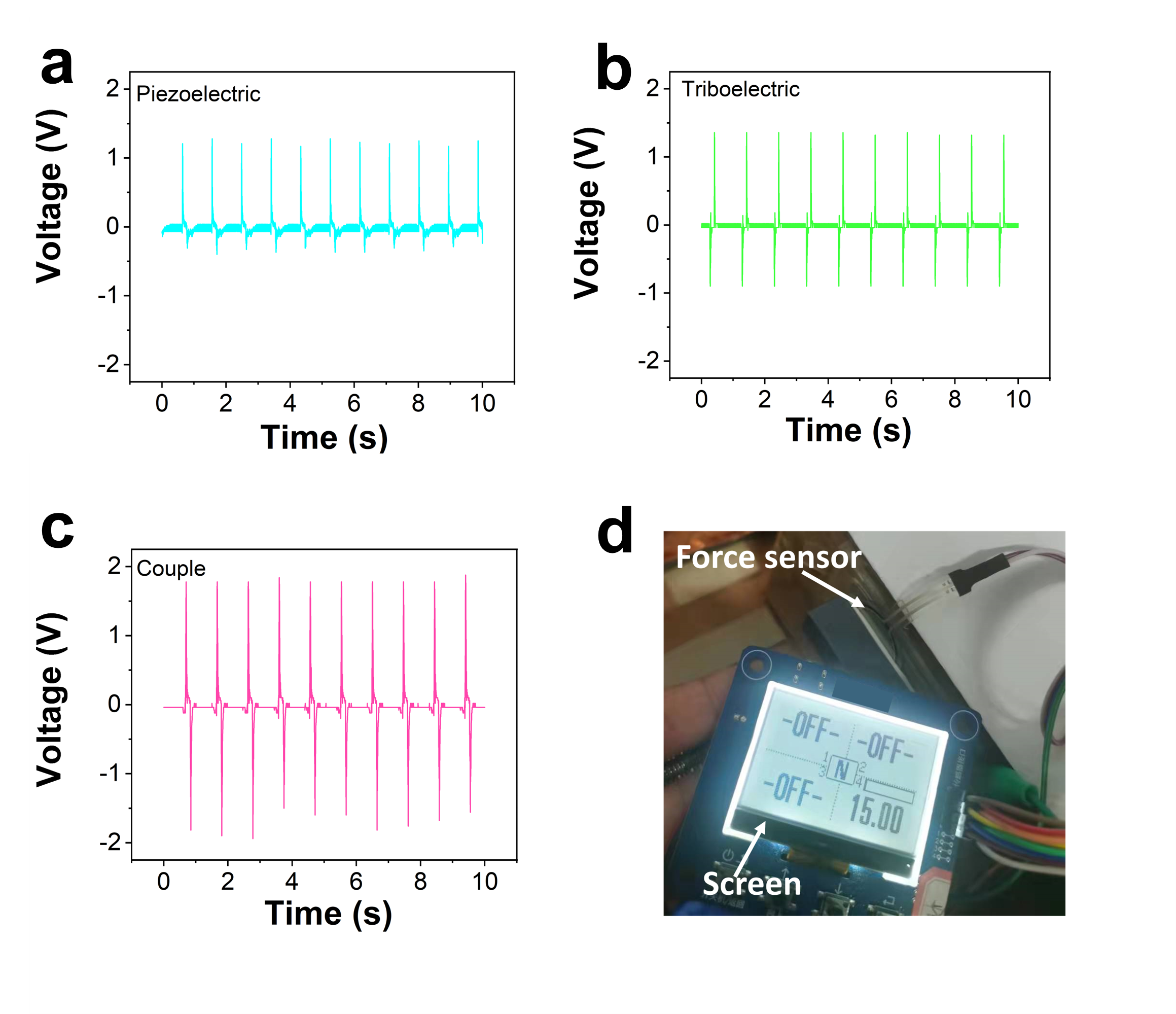

Supplement: Supplementary file 1 [file nanomaterials-12-00104-s001.zip › nanomaterials-1491178/nanomaterials-1491178-Figure , Movie and SI-R3/Fig S3.tif]

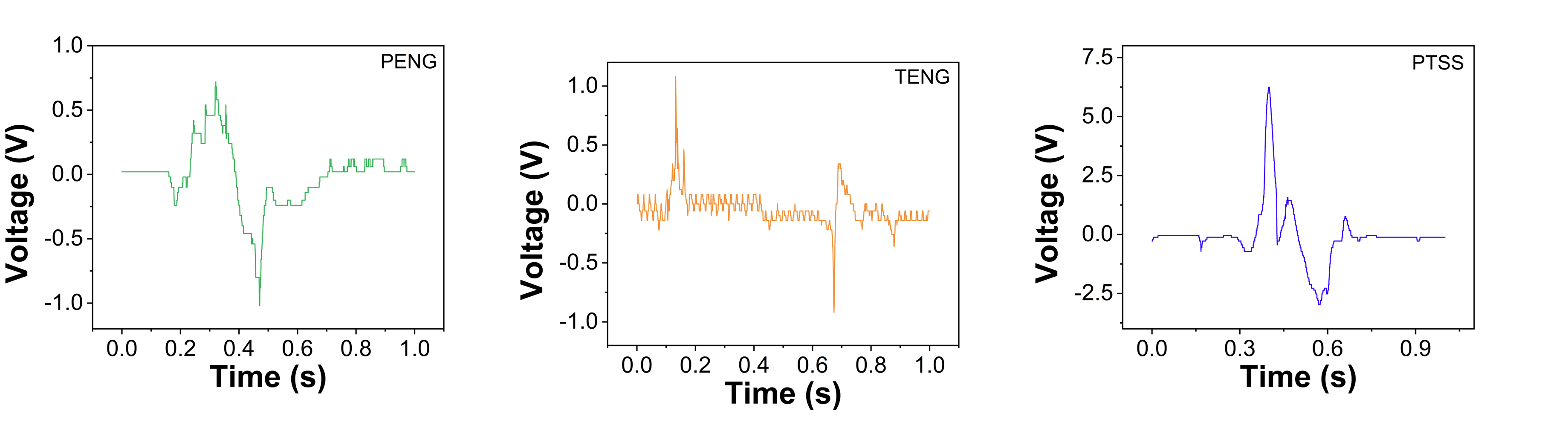

Supplement: Supplementary file 1 [file nanomaterials-12-00104-s001.zip › nanomaterials-1491178/nanomaterials-1491178-Figure , Movie and SI-R3/Fig S4.tif]

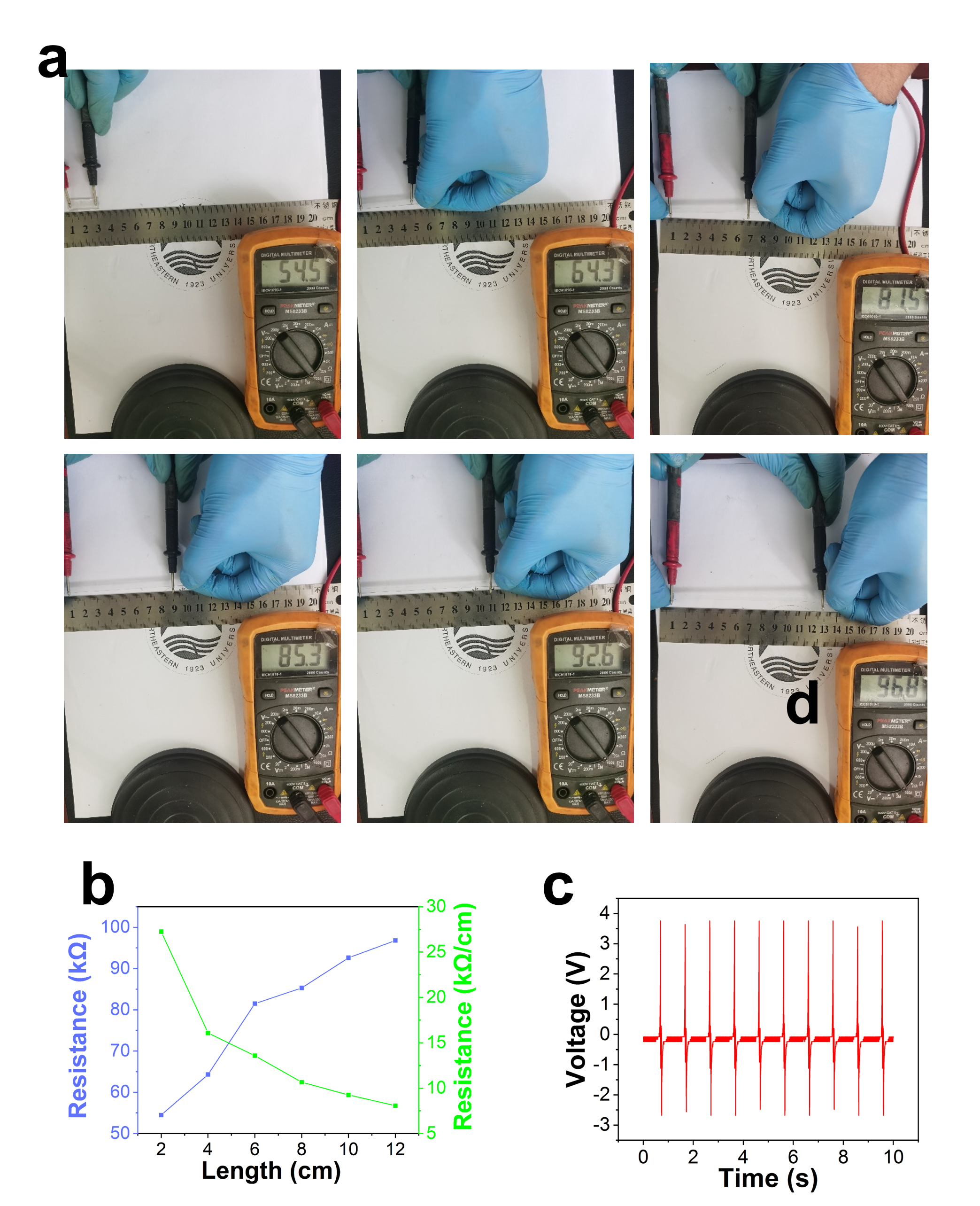

Supplement: Supplementary file 1 [file nanomaterials-12-00104-s001.zip › nanomaterials-1491178/nanomaterials-1491178-Figure , Movie and SI-R3/Fig S5.tif]

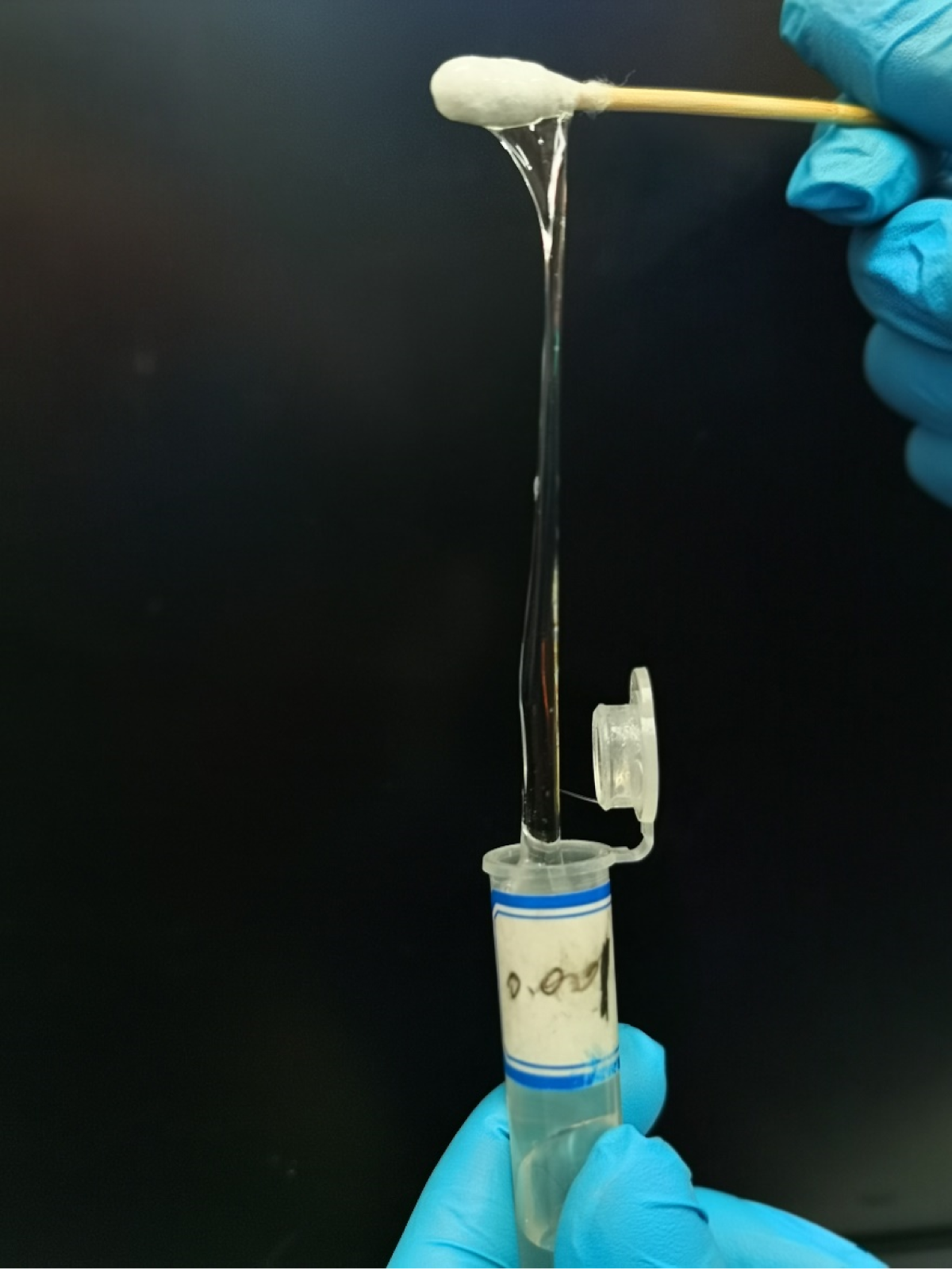

Supplement: Supplementary file 1 [file nanomaterials-12-00104-s001.zip › nanomaterials-1491178/nanomaterials-1491178-Figure , Movie and SI-R3/Fig S6.tif]

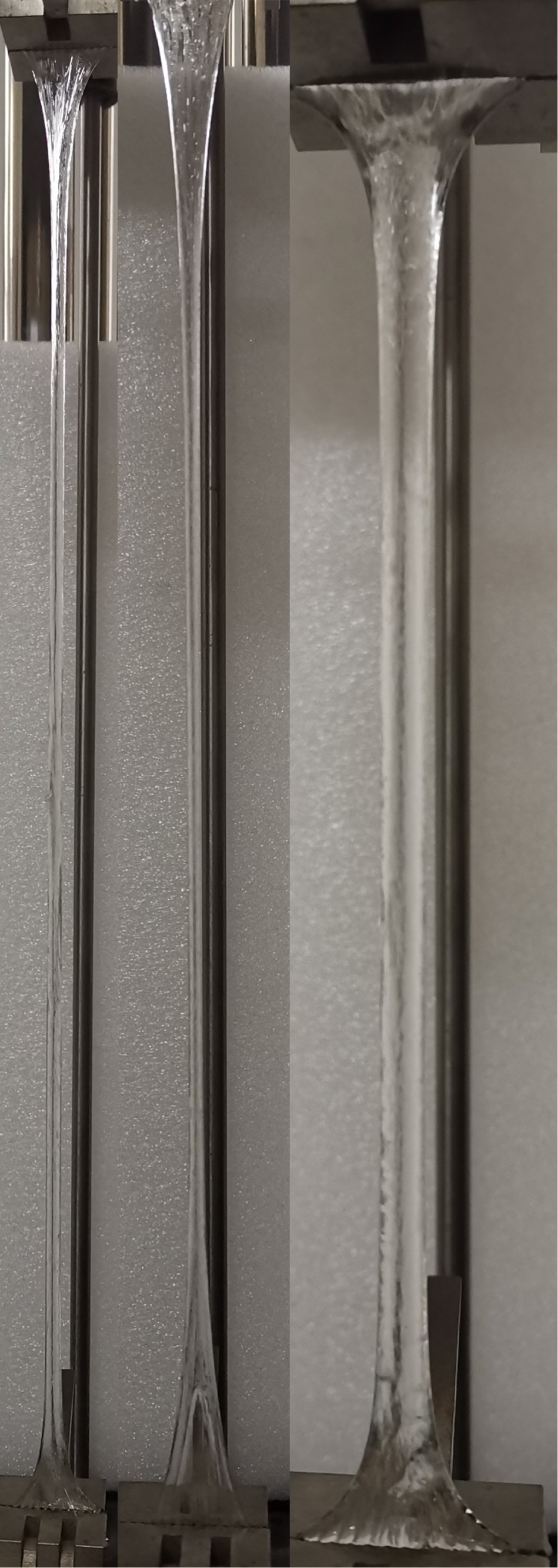

Supplement: Supplementary file 1 [file nanomaterials-12-00104-s001.zip › nanomaterials-1491178/nanomaterials-1491178-Figure , Movie and SI-R3/Fig S7.tif]

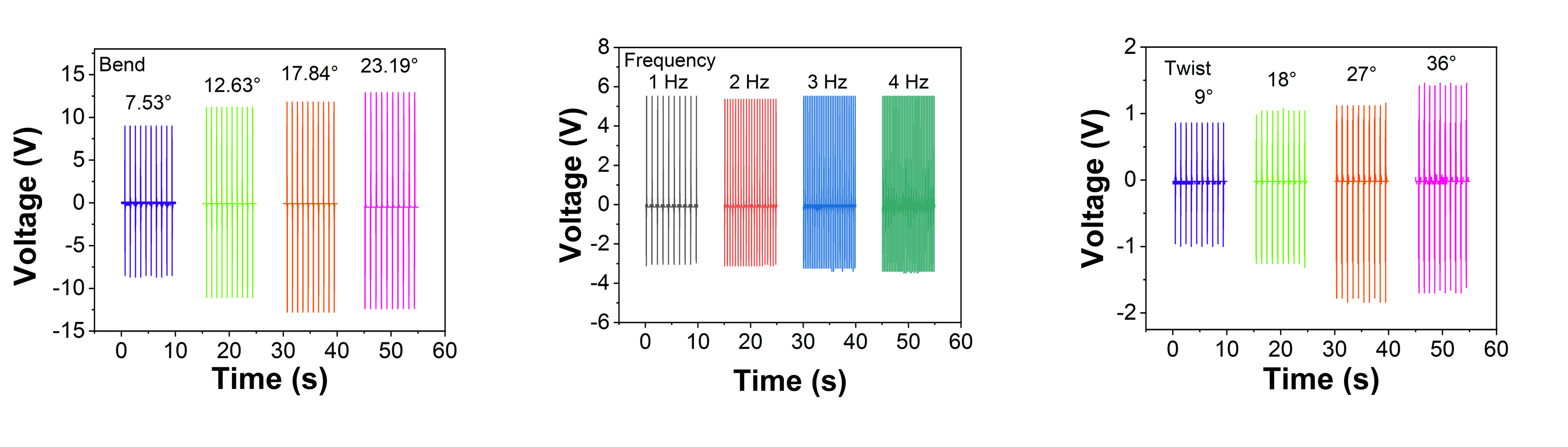

Supplement: Supplementary file 1 [file nanomaterials-12-00104-s001.zip › nanomaterials-1491178/nanomaterials-1491178-Figure , Movie and SI-R3/Fig S8.tif]

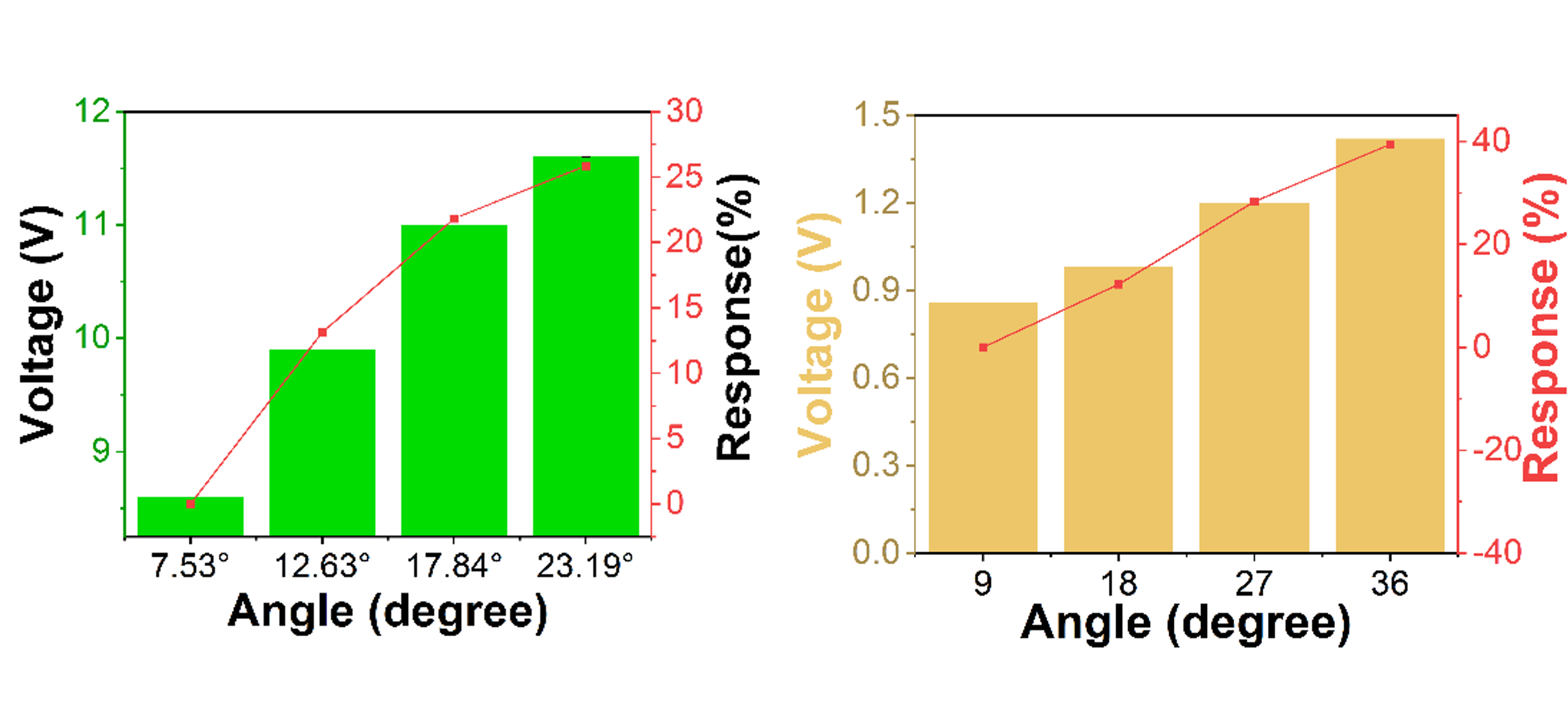

Supplement: Supplementary file 1 [file nanomaterials-12-00104-s001.zip › nanomaterials-1491178/nanomaterials-1491178-Figure , Movie and SI-R3/Fig S9.tif]
